# The Complete Mitochondrial Genome of an 11,450-year-old Aurochsen (*Bos primigenius*) from Central Italy

**DOI:** 10.1186/1471-2148-11-32

**Published:** 2011-01-31

**Authors:** Martina Lari, Ermanno Rizzi, Stefano Mona, Giorgio Corti, Giulio Catalano, Kefei Chen, Cristiano Vernesi, Greger Larson, Paolo Boscato, Gianluca De Bellis, Alan Cooper, David Caramelli, Giorgio Bertorelle

**Affiliations:** 1 Dipartimento di Biologia Evoluzionistica, Università di Firenze, Firenze, Italy; 2 Istituto di Tecnologie Biomediche, CNR, Segrate (MI), Italy; 3 Dipartimento di Biologia ed Evoluzione, Università di Ferrara, Ferrara, Italy; 4 Australian Centre for Ancient DNA, University of Adelaide, Adelaide, Australia; 5 IASMA Research and Innovation Centre, Fondazione Edmund Mach, Trento, Italy; 6 Department of Archaeology, Durham University, Durham, UK; 7 Dipartimento di Scienze Ambientali, Università di Siena, Siena, Italy; 8 EPHE (Ecole Pratique des Hautes Etudes), Muséum National d'Histoire Naturelle, Paris, France

## Abstract

**Background:**

*Bos primigenius*, the aurochs, is the wild ancestor of modern cattle breeds and was formerly widespread across Eurasia and northern Africa. After a progressive decline, the species became extinct in 1627. The origin of modern taurine breeds in Europe is debated. Archaeological and early genetic evidence point to a single Near Eastern origin and a subsequent spread during the diffusion of herding and farming. More recent genetic data are instead compatible with local domestication events or at least some level of local introgression from the aurochs. Here we present the analysis of the complete mitochondrial genome of a pre-Neolithic Italian aurochs.

**Results:**

In this study, we applied a combined strategy employing both multiplex PCR amplifications and 454 pyrosequencing technology to sequence the complete mitochondrial genome of an 11,450-year-old aurochs specimen from Central Italy. Phylogenetic analysis of the aurochs mtDNA genome supports the conclusions from previous studies of short mtDNA fragments - namely that Italian aurochsen were genetically very similar to modern cattle breeds, but highly divergent from the North-Central European aurochsen.

**Conclusions:**

Complete mitochondrial genome sequences are now available for several modern cattle and two pre-Neolithic mtDNA genomes from very different geographic areas. These data suggest that previously identified sub-groups within the widespread modern cattle mitochondrial T clade are polyphyletic, and they support the hypothesis that modern European breeds have multiple geographic origins.

## Background

Genomic analyses of ancient samples are limited principally by DNA preservation. Standard ancient DNA methods that consist of amplification, followed by cloning and sequencing of multiple clones, have been used to obtain mitochondrial genomes from the bones of mammoths and other permafrost-embedded animals, where up to 400-500 base pair DNA fragments can be retrieved [1-4]. However, these methods are not as useful for less well-preserved samples [5] where the preference is for different approaches based on the development of metagenomic libraries or direct large-scale genome sequencing through Next Generation massively-parallel sequencing. For example, the mitochondrial genome and several million base pairs of nuclear DNA from Neanderthal bone fragments were sequenced with a Next Generation approach [6-8] and 80% of the diploid genome from an extinct Paleo-Eskimo was recovered with a similar procedure [9]. These powerful technologies are extremely well suited for the analysis of bulk genomic DNA extracted from ancient remains [6,10,11] but their use for characterization of the mitochondrial genome is less effective outside of mtDNA-enriched tissues such as hair shafts [12-15]. Recently, selective target enrichment prior to Next Generation ultra-deep sequencing has also been shown to be an appropriate method for the characterization of mitochondrial genomes from ancient tissues [16-20].

**Figure 1:**
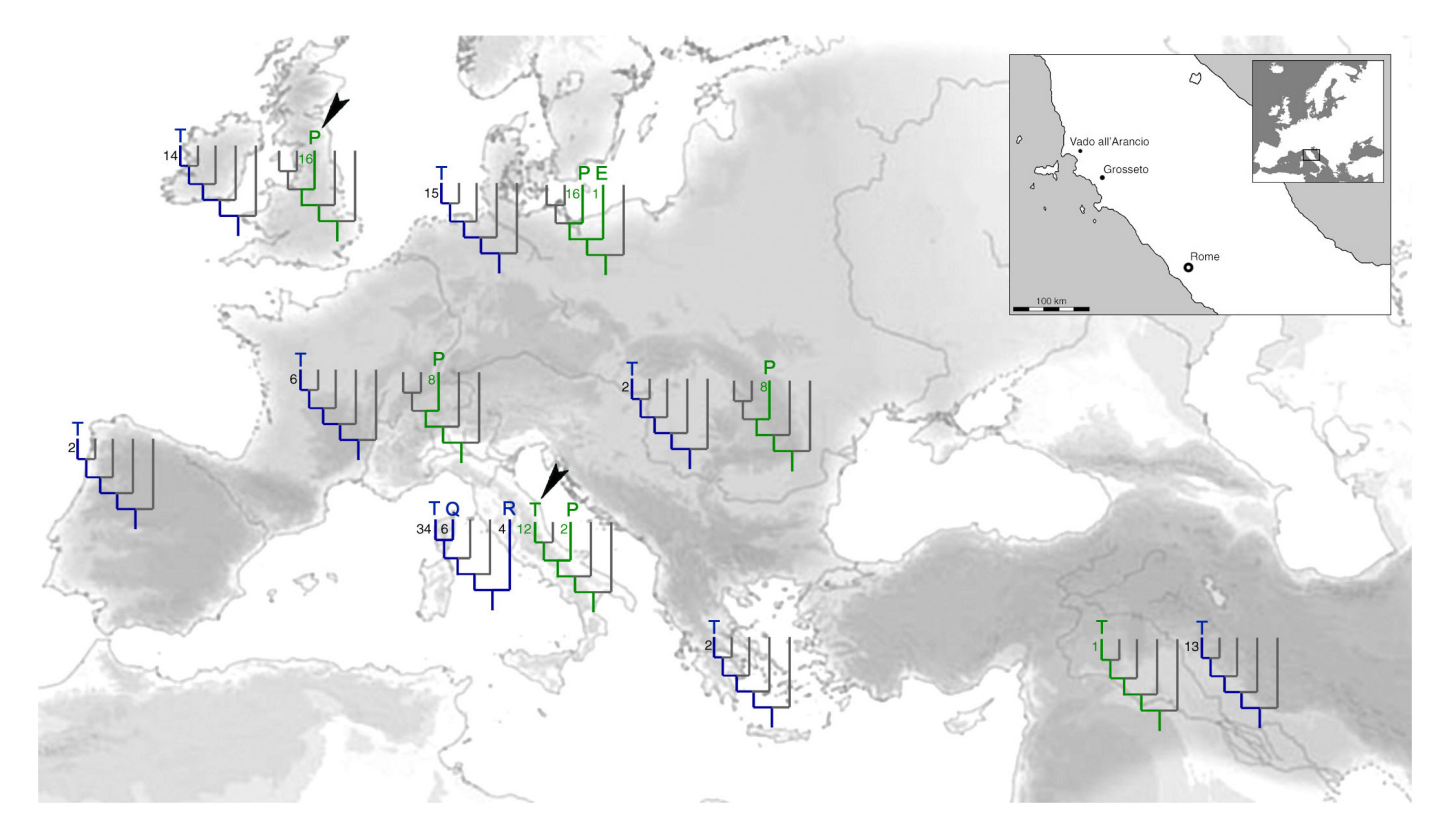
**Geographical distribution of mtDNA major clades**. Mitochondrial D-loop sequences in ancient aurochen are reported as green branches on the phylogenies, with the number of separate individuals indicated, along with the current lineage nomenclature (P, E and T). Complete mtDNA genomes in modern cattle breeds are reported as blue branches (lineages T, Q and R) and similarly numbered. The phylogenetic affiliation of the available aurochen mtDNA genomes ([30]; this study) are indicated by the two black arrows. The geographic location of the Vado all'Arancio site is indicated in the figure inset.

In this study, we applied a combined strategy that made use of multiplex PCR amplifications and 454 pyrosequencing technology to sequence the complete mitochondrial genome of a *Bos primigenius *sample excavated from Vado all'Arancio rockshelter in Central Italy (see inset in Figure 1), dated by associated remains at around 11,450-years. *Bos primigenius*, the aurochs, is the wild ancestor of modern cattle breeds and was formerly widespread across Eurasia and northern Africa. After a progressive decline thought to be due to overhunting and habitat contraction, the species became extinct in 1627. The history of cattle domestication and the degree of genetic contribution of local aurochsen to modern taurine breeds in Europe is still a matter of debate [21-30]. While previous studies have utilised both modern and ancient DNA sequences, the ancient data consisted almost exclusively of short fragments of the mitochondrial control region. These studies suggested that all Northern and Central European aurochsen and a small fraction of Italian aurochsen had control region sequences belonging to haplogroup P [29], whereas the typical Italian aurochsen belonged to haplogroup T [24,29]. Modern taurine cattle also possess haplogroup T, with the exception of a handful of individuals who have sequences attributed to the aurochs haplogroup P, or the putative aurochs haplogroups R and Q (Figure 1). Recently, the first aurochs mtDNA genome was typed from a 6,700-year-old bone sample located in England [30], and this sequence was found to belong to haplogroup P, consistent with the results from the short control region sequences. The present study reports the first pre-Neolithic aurochs mitochondrial genome typed from Southern Europe, and confirms the view that the aurochs was genetically structured in Europe, with different local populations having different genetic relationships with the modern cattle.

## Results and Discussion

### The Bos primigenius mtDNA genome

The combined multiplex PCR and 454 sequencing procedure generated more than 85,000 total reads from the Vado all'Arancio aurochs phalanx bone. Approximately 66% of the reads were mapped to the bovine reference mtDNA sequence (BRS) [31]. After excluding false insertions and deletions commonly introduced by the 454 sequencing technology at homopolymeric strings, a total of 7,565,547 bases (Table S1, Additional File 1) were used to assemble a preliminary consensus sequence. The frequency distribution of the number of reads per nucleotide (Figure S1a, Additional File 2) is irregular, due to the overlap of fragments and because specific fragments were pyrosequenced more than once. The mean and median number of reads per nucleotide were 463 and 93 respectively. Overall, the number of reads for each specific fragment analysed with the 454 approach was between one and two orders of magnitude higher than the number of clones commonly sequenced using traditional aDNA approaches. At each site, the most frequent nucleotide was observed in 99.4% of the reads on average (Figure S1b, Additional File 2).

Among the 4113 sites where amplicons overlap, we analysed if different error patterns were observed at sites which are either monomorphic or polymorphic in modern breeds, or at homologous sites typed in different amplicons. Different patterns are expected in case of contamination with modern DNA. Very similar allele frequencies spectra were observed considering separately monomorphic and polymorphic sites in modern breeds, and only 6 out of 176 sites which are polymorphic in the cattle had a rare allele at a frequency > 2% among the reads (and never larger than 10%). In addition, the frequency of nucleotide misincorporation among reads shows a pattern typical of ancient templates, with an excess of type II over type I transitions (ratio typeII/typeI = 1.58; see Table S2, Additional File 3).

The Vado all'Arancio aurochs sequence showed eight indels and nine mutations compared to the *Bos taurus *reference sequence [31]. However we noted that all but one of the indels involved an insertion/deletion of the last base in a homopolymeric string of > 5 bases, which is likely to result from an artefact of the 454 pyrosequencing technology [32]. In two other positions, C- > T substitutions were present in only 54% and 58% respectively of the total reads, a strong signal of nucleotide misincorporations due to degradation of the original templates rather than real substitutions. For this reason we carefully checked these ambiguous positions by singleplex PCRs, cloning and Sanger sequencing (Figure S2, Additional File 4) before assembling the definitive consensus sequence for the specimen of 16,339 bp.

The consensus Vado all'Arancio aurochs sequence differed from the *Bos taurus *reference sequence (BRS) at just four transitions, three transversions and one insertion, all located in the mtDNA coding region (Table 1). The positions were confirmed independently in three different aDNA labs (Table S3, Additional File 5 and Figure S3, Additional File 6), according to standard practice. When compared to 134 complete mtDNA sequences from different cattle breeds [27,28], only three of these positions (12744, 14159, 15384) correspond to fixed differences. On average, the Vado all'Arancio aurochs mtDNA genome differs from modern domestic cattle at 15.2 sites (range: 5 - 86), a value similar to the average distance between two modern domestic cows (16.6 sites, range: 0 - 97). 73 differences were observed between our sample and the complete genome of the 6,700-year-old English sample [30]. Substitutions are distributed in the non-coding control region (30.14%), rRNA and tRNA genes (16.44%) and in 11 protein genes (53.42%) (Table S4, Additional File 7).

**Table 1:** Mutations in the Italian *Bos primigenius *mtDNA genome compared to the Bovine Reference Sequence (BRS) [31]

Position	BRS	*Bos p*.	Region
587	-	+C	rRNA 12S
2536	C	A	rRNA 16S
9682	G	C	COX3
12738	C	T	ND5
12744	C	T	ND5
13310	A	C	ND5
14159	A	G	ND6
15384	G	A	CYTB

### Phylogenetic analysis

**Figure 2:**
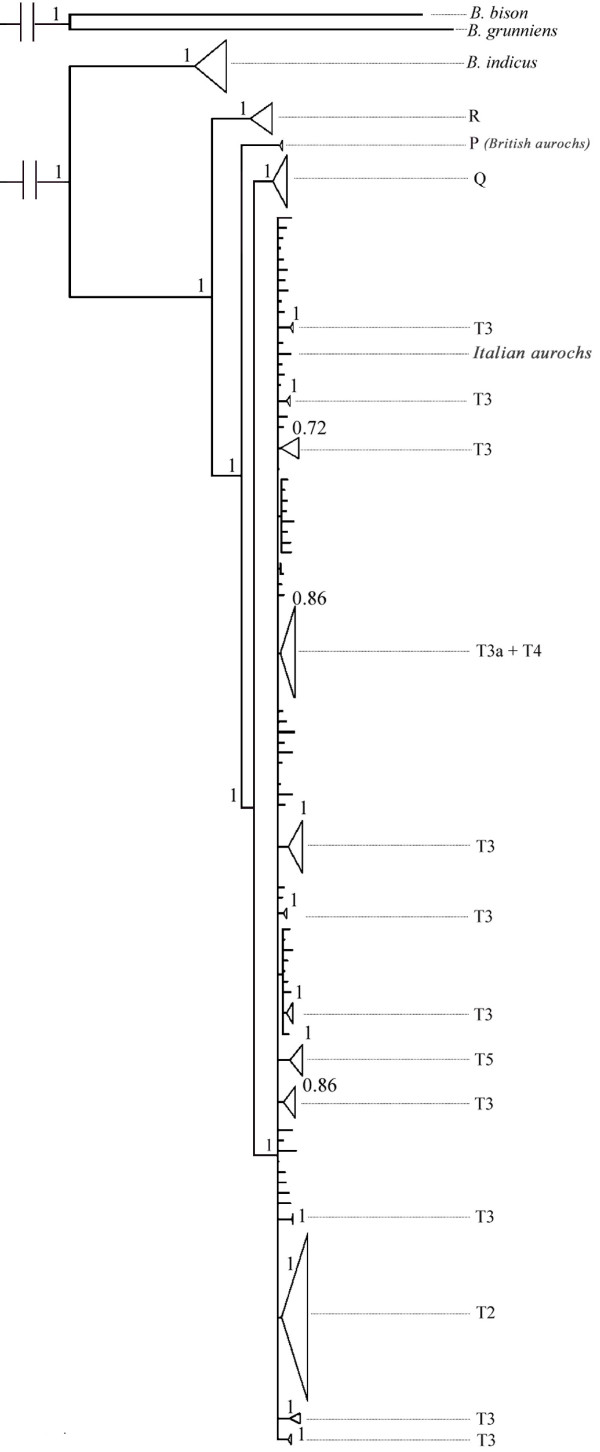
**Phylogenetic tree of complete mtDNA genomes**. Bayesian consensus phylogenetic tree produced by PHYCAS under a prior model allowing for polytomies. Clusters of sequences linked by posterior probabilities higher than 0.7 have been collapsed. Sequences belonging to cluster T are not collapsed in order to show sub groupings, and the traditional haplogroup nomenclature is shown on the right. Clades R, P, Q and T are monophyletic, but only subclades T2 and T5 are supported as definable groups amongst the previously recognized T subclades. The disparate phylogenetic positions of the Italian and the British aurochsen are indicated. All other tips refer to modern cattle genomes.

Bayesian phylogenetic analysis of the European cattle and aurochs mtDNA coding genomes revealed that a model allowing for polytomies is strongly supported over a strict bifurcating model (Bayes Factor > 100). Therefore, the pattern of previously classified bovid clades and sub-clades is not supported, suggesting that recurrent mutations and short internal branches may limit meaningful evolutionary information. Overall, the structure of the Bayesian consensus tree (Figure 2) is similar to the tree reported in [27] and [33], but only the major clades (R, P, Q, and T) and two subclades (T2 and T5) are monophyletic. This result is supported by analysis of the mtDNA control region [33] (this study, results not reported). This suggests that the previously identified T1, T3 and T4 clades do not correspond to robust evolutionary clades, and suggests that evolutionary inferences in cattle and aurochs based on mtDNA sub-clades frequencies should be avoided.

The Vado all'Arancio aurochs mtDNA genome clearly belongs to the major clade T, and is embedded within the European cattle mitochondrial genealogy in contrast to the British aurochs specimen [30], as previously suggested [24,29]. Polymorphisms within the control region sequence of the Vado all'Arancio specimen had previously led to it being assigned to the T3 sub-haplogroup [24]. However, our statistical analysis of complete genome sequences indicates that T3 is not monophyletic and is therefore not a useful designation for inferring evolutionary processes. In addition, the Vado all'Arancio sequence does not cluster with any of the cattle sequences previously assigned to the T3 sub-haplogroup (see Figure 2).

### Demography, domestication and clades dating

**Figure 3:**
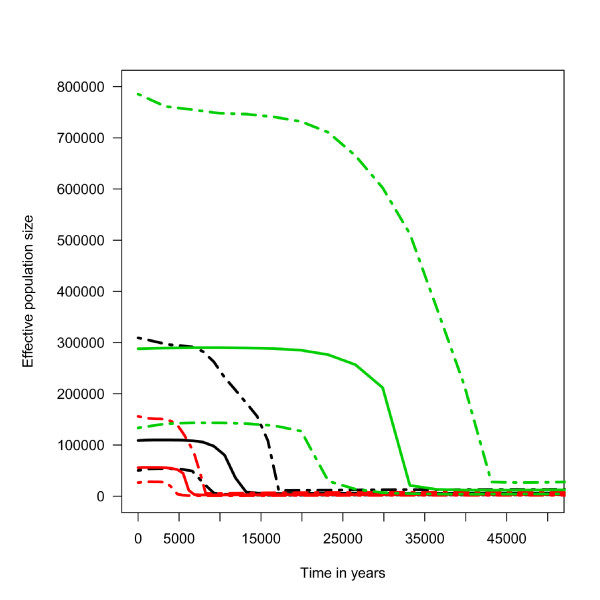
**Bayesian skyline plot**. Bayesian skyline plot constructed using the Italian cattle dataset with the *Bos primigenius *sample under three different evolutionary rates 3.3*10^-8^, red, based on [35]; 1.6*10^-8^, black, based on [27]; 6.6*10^-9^, green, based on [36]. The continuous lines represent the median estimates; dotted lines represent the 95% HPD interval.

A Bayesian skyline analysis was performed with BEAST [34] to estimate the demographic history of Italian cattle. 44 modern mtDNA genomes from previous studies [27,28] were used for this analysis. We selected this geographically homogenous subset of mtDNA genomes to reduce possible biases due to population structure. As expected from previous studies of the mtDNA control region in European cattle [33], a strong signal of rapid demographic expansion was inferred (Bayes Factor > 20 when compared to a constant size model) (Figure 3). The estimated starting date of this expansion varies between about 7,000 and about 35,000 years BP depending on the calibration date used for the divergence time between *B. taurus *and *B. bison*. In fact, at least three dates have been proposed for this split: 1 MYA [35], 2 MYA [27] and 5 MYA [36]. The rapid increase in population size inferred by the Bayesian skyline analysis also points to a very small effective size prior to the expansion, which is compatible with the long-term population size estimated for several modern cattle breeds [37].

It seems therefore that the inferred demographic expansion corresponds to the rapid increase of cattle numbers occurred less than 10,000 years ago during the Neolithic domestication process, considering also that no evidence of expansion was found when pre-domestication specimens were analysed [29]. If correct, this would suggest that the most likely divergence time between *B. taurus *and *B. bison *is older than 1 MYA but much younger than 2 MYA.

We used the 1 MYA calibration date to estimate the age of several monophyletic mtDNA haplogroups in the reconstructed phylogeny (see Table 2), although it is important to note that these dates are only rarely related to population events (see for example, [38]). The TMRCA (time to the most recent common ancestor) in this species is slightly younger than 100 thousand years, while the origin of the most frequent clade observed in domestic European breeds (haplogroup T) clearly predates the Neolithic revolution. This result is compatible with our finding that a pre-Neolithic aurochs also had a T sequence. For comparison, the TMRCA ages for the same clades were calculated with a 2 MY calibration point, and compared with results from [28], where the 2MY calibration point and simpler statistical methods (based either on a fixed topology or on the pairwise estimator *Rho) *were used. As expected from theoretical arguments [39], we found that these simple methods tend to underestimate the clade ages.

**Table 2:** Haplogroups age estimation

	Age assuming a 1 MY calibration point (95% HPD)	Age assuming a 2 MY calibration point (95% HPD)	Age computed in Achilli et al. 2009 under a fixed topology model
RPQT	83.2 (68.2 - 99.0)	166.9 (138.0 - 197.4)	135.3 (13.1)
PQT	45.6 (35.8 - 55.7)	91.4 (72.1 - 111.7)	70.8 (8.8)
QT	34.4 (26.8 - 42.5)	69.1 (54.1 - 85.6)	48.2 (7.2)
T	18.3 (14.4 - 22.5)	36.5 (28.7 - 45.5)	15.8 (3.1)
T2	12.6 (9.4 - 16.1)	25.2(18.5 - 31.6)	10.4 (0.9)
T5	8.8 (3.8 - 14.3)	17.2 (7.2 - 28.5)	10.2 (3.0)

The estimated age (median and 95% HPD of the posterior distribution, in thousands of years) of several key monophyletic clades was calculated assuming that the *B. taurus *- *B. bison *divergence occurred 1 MYA [35]. This calibration point is compatible with the demographic expansion detected in cattle mitochondrial data resulting from the Neolithic domestication event. Ages are also calculated assuming that *B. taurus *- *B. bison *diverged 2 MY [27]. For comparison, the last column reports the maximum likelihood estimates obtained in a previous study based on the 2 MY calibration point but a fixed topology [28], with standard errors in parenthesis.

Finally, we investigated the effect of adding the Italian aurochs genome on the TMRCA estimates for mtDNA clades, by reanalyzing the data with and without the aurochs sequence. The aim of this analysis is to understand how the addition of this single ancient sequence influences the estimates of the timing of the origin of each clade in the genealogy. We assumed genealogical continuity between ancient and modern samples and applied the single population flexible coalescent model (Bayesian skyline) implemented in BEAST. Under this model, ancient DNA sequences from specimens with known age can be appropriately included in the data set, and given the phylogenetic position of the aurochs genome (Figure 2) this analysis appears robust to deviations from the single population assumption. The inclusion of the aurochs genome increased the estimated age of the T clade from 12.1 KY (HPD 95%: 8.2 - 16.4) to 16.1 KY (HPD, highest posterior densities, 95%: 12.7 - 19.8). Thus, the inclusion of an 11 KY-old T haplotype sequence increases the estimated TMRCA of the relatively recent T clade by about one third under a coalescent population model. The 16.1 KY value is compatible with the age estimated from the full European mtDNA genome dataset and the phylogenetic approach. As expected, the addition of the aurochs genome has little impact on the estimated TMRCAs for the more basal clades. For example, the median TMRCA for RPQT and QT estimated with the aurochs genome are 83.6 KY (95% HPD: 68.0 - 101.1) and 32.5 KY (95% HPD: 24.0 - 41.3) respectively, and 82.8 KY (95% HPD: 66.7 - 100.1) and 30.1 KY (95% HPD: 21.8 - 40.0) without it.

Clearly, more ancient genomes will be necessary to fully investigate the role of aurochs in the crucial time interval following the domestication of cattle. However, the data available so far suggest that the genetic variation in the cattle was strongly affected by a bottleneck during the domestication process followed by an intense demographic expansion, while a constant population size model appears more appropriate for the aurochs dynamic [29].

## Conclusions

The complete *Bos primigenius *mtDNA genome generated from an 11 KY Italian skeleton as part of this study has a genetic distance of 0.45% from a 6,700-year-old sample recovered in England [30]. While the British genome belongs to the now extremely rare P clade, the Italian genome belongs to the T clade, a pre-Neolithic homogeneous group of sequences which contains sequences from the vast majority of modern cattle. This supports the view, previously based on short mitochondrial fragments from several Mesolithic individuals and modern breeds, that aurochs populations in northern and southern regions were clearly differentiated, and that southern European aurochsen may have played a role during the domestication of modern cattle [24,28,29]. Using a Bayesian approach, we also show that the domestication process left a significant trace of rapid demographic expansion in cattle mtDNA genomic variation. Finally, our phylogenetic analyses suggest that efforts to assign aurochs mitochondrial genomes within specific cattle sub-clades of the T clade may be irrelevant, since the branching pattern within this clade appears to contain several polytomies. This suggests that studies of the domestication process, which essentially involved individuals bearing T mtDNA genomes, may be particularly complicated, and are likely to require additional information from nuclear markers.

## Methods

### Mitochondrial genome sequencing in the aurochs

Various preliminary biochemical tests indicated that a phalanx bone sample excavated in Vado all'Arancio rock shelter (Massa Marittima, Grosseto, Italy, Figure 1) and ascribed to *Bos primigenius sp*. by morphological and morphometrical analysis [40], was well-preserved for molecular analysis (Table S5, Additional File 8 and Figure S4, Additional File 9). Three lines of evidence suggested that endogenous biomolecules were likely to be well-preserved [41,42]. Firstly, the degree of racemization for three amino acids (aspartic acid, alanine and glutamine) was low (Table S5, Additional file 8), and this has been suggested to be compatible with DNA survival [42,43], though concerns have been raised about the utility of this approach [44,45]. Second, we calculated that, under our reaction conditions, the estimated copy number of a 180 bp mtDNA target was greater than 5000 copies per reaction (5127 ± 912; standard curve fit values: Slope = -3.391, Y-intercept = 45.34, R^2 ^= 0.977, Efficiency = 0.97). This value is much larger than the threshold under which consensus sequence determination is thought to be seriously affected by nucleotide modifications present in the ancient DNA molecules [46]. Third, using a system of three overlapping primer pairs we obtained the same sequence for the hypervariable segment of the mtDNA control region from different bone fragments and in multiple PCRs (Figure S4, Additional File 9).

The Vado all'Arancio rock shelter is a well known Italian site with a clear stratigraphy, and was occupied only for a short period of time during the Late Palaeolithic. The presence of specific artifacts and the radiocarbon dating of associated faunal remains (11,300 ± 150BP obtained in Rome for R1333, and 11,600 ± 130 obtained in Lyon for Ly3415; average of 11,450 years BP) unequivocally date Vado all'Arancio rockshelter to a pre-Neolithic context preceding the spread of domestication [40]. This further confirms that the specimen belongs to an aurochs, rather than an early Holocene *Bos taurus*.

We applied a combined typing strategy for mtDNA genome sequencing. Multiplex PCR amplifications of short overlapping fragments covering the whole mtDNA genome were used to enrich the total extracted DNA, prior to 454 pyrosequencing of the pooled enriched material. A multiplex PCR approach has previously been used for the reconstruction of ancient mithocondrial genomes [1,47,48,17]. Compared with a singleplex PCR strategy, multiplex PCR has two advantages: first, a small quantity of starting material can be used to retrieve multiple DNA fragments simultaneously, and second, limited manipulation of the original template reduces the risk of sporadic contamination by exogenous DNA.

We designed 130 overlapping primer pairs on the basis of *Bos taurus *reference sequence (BRS) [31]. Primer pairs (designed to produce 155 to 230-bp long DNA sequences) were arranged into four sets (A,B,C,D) with overlapping pairs in different sets (see Additional File 10). Each multiplex PCR set was performed on each different extract following stringent criteria for ancient DNA analysis and sequence authentication [41]. The amplification success for each primer pair was checked with secondary singleplex PCRs. All the amplification products were then diluted to equal concentration, pooled, and used as a substrate for the FLX library preparation and pyrosequencing reaction using the Roche FLX/454 technology. After identifying sequence reads with the PCR primers at their termini, the primers were masked and the resulting portion mapped against the reference sequence [NCBI accession: V00654] using the Amplicon Variant Analyzer application (AVA, Roche) with default parameters. Finally, starting from the AVA multi-alignments, we generated consensus sequences using a home-made Python script, which assigned the most frequent base at each position. A large number of mtDNA amplicons were sequenced to allow the consensus sequence to be determined accurately, without laborious cloning and sequencing of PCR products. Amplicons with no or low coverage (< 10 reads) after the first round of sequencing were pooled again and pyrosequenced separately.

Standard ancient DNA singleplex PCRs, cloning and Sanger sequencing approaches were used to fill remaining gaps and resolve ambiguous sequences after the first assembly of the total reads generated with the 454 technology. Particular attention was paid to insertions or deletions in homopolymer regions that are problematic for the 454 pyrosequencing technology [32], and to potential misincorporations due to degradation in the original template (see Additional File 10).

Finally, to check the reproducibility of the results, we replicated 16 selected amplicons in three different laboratories in Italy, Sweden and Australia (see Table S3, Additional File 5, and the Additional File 10). Amplicons that showed polymorphic positions with respect to the BRS sequence, or with critical phylogenetic markers, were replicated at Uppsala University (Sweden) and at the Innovation and Research Centre (San Michele all'Adige, Trento, Italy). Control region fragments and a small portion of the 12S gene were sequenced at Adelaide University (Australia).

Additional details regarding the sample and the molecular procedures are reported in the Additional File 10.

### Phylogenetic analysis and demography

Phylogenetic inferences were performed on the coding region (15440 bp) of the mtDNA alignment. The model of nucleotide substitution was selected by means of the MODELTEST software [49], according to the Akaike information criterion (AIC). The model resulting with the lowest AIC score was the GTR+γ+I (general time reversible model with heterogeneity in substitution rates plus a proportion of invariable sites). We performed Bayesian phylogenetic inference under two prior models: *a) *an unrooted, strictly bifurcating model, and *b) *an unrooted model allowing for polytomies (following the algorithm proposed in [50]). Both analyses were performed using the software PHYCAS [51], and the input file for this analysis is provided as Additional File 11.

The first model is similar to the standard unrooted model implemented in MRBAYES 3.1.2 [52] with the difference that a hyperprior parameter is used to model the rate of the exponential prior distribution on branch lengths (while in MRBAYES such parameter is fixed and set by the user). We used as hyperprior distribution an inverse gamma with mean 1.0 and variance 10.0, following [53]. In the second model, a *polytomy prior *[50] needs to be set. This value indicates the relative strength of a less resolved topology compared to a more resolved one: a value equal to 1 gives same prior to polytomic or strictly bifurcating topologies, while values greater than 1 places more weight on polytomic topologies. We set the *polytomy prior *to *e *(Nepero's number), following [50]. PHYCAS uses slice sampling to update continuous parameters and the LOCAL move of Larget and Simon [54] to update topology and branch lengths. In the second model that allowed for polytomies, a RJ-MCMC scheme was used because each iteration the number of parameters can change (as the number of branches vary due to the presence of polytomies).

PHYCAS was run twice for 200,000 cycles for both models. A cycle on PHYCAS roughly corresponds to 100 iterations in MRBAYES. We sampled one cycle every 100, after discarding the first 100,000 cycles as burn-in. We compared the traces of the two runs to check for convergence. After the burn-in was removed, we compared both models using Bayes Factors. The Bayes Factor was computed as twice the difference between the log of the marginal likelihoods, which were approximated using the harmonic mean as suggested in [55]. To check if the heating procedure could produce different results (which would in turn implies that a single MCMC chain could not converge properly to the correct posterior distribution), we ran the first model using MRBAYES. The analysis was run twice using four incrementally heated chains with the default temperature, over 2*10^8 ^generations long with a 20% burnin. Convergence was checked by examining the generation plot visualized with TRACER [56] and we computed the potential scale reduction factor [57] with the sump function in MRBAYES. The resulting tree topology as well as the posterior probabilities of the various nodes were almost identical to the results obtained with PHYCAS, suggesting that the posterior distribution was correctly explored using PHYCAS even without the heating procedure.

Molecular dating is subject to many sources of errors. Indeed, usually topology and branch lengths are not known a priori and they need to be estimated from data with the associated uncertainty in the estimation procedure. In addition, the calibration of molecular clock relies on a known divergence time which is often assumed as a fixed value. The importance of a correct calibration point (usually a fossil divergence) has been well acknowledged in the phylogenetic literature, though it is difficult to obtain a reliable estimate that can be readily translated into an accurate molecular clock rate. The *Bos *spp. phylogeny is no exception to this rule, and several different paleontological data have been used to calibrate the bovid mitochondrial clock. For this reason, we employed three divergent, though widely used calibration points and performed the following analyses for all of them. The three points were: a split between *B. taurus *and *B. bison *at 1 MYA [35], at 2 MYA [27] and at 5 MYA [36].

We first performed phylogenetic dating on all our dataset employing a bayesian algorithm implemented in BEAST [58]. The input file for BEAST is provided as Additional File 12. We choose a Yule prior [59] on topology and branch lengths and constrained the root of our phylogeny (which coincides with the split *B. taurus-B. bison) *to one of the three calibration points above (i.e., 1MY, 2MY, and 5MY). Since the new *B. primigenius *specimen is dated at more than 11,000 years B.P., we excluded it from this analysis as the Yule prior has not been adapted to handle serial data. We ran each of these analyses twice for 20,000,000 MCMC, with a thinning value of 1,000. Since these models differ only in the age of one node, we could have just estimated the scaled branch lengths and used the three calibration points to translate them into years. The three analyses can be however considered as an additional check for convergence. We removed the first 10% MCMC iterations as burnin. Under this phylogenetic dating approach, we computed the TMRCA of several nodes of interest and, at the same time, estimated the posterior distribution of the molecular clock rate.

We then selected only Italian cattle and performed bayesian based population genetic inference and molecular dating under two coalescent priors: constant population size and the Bayesian skyline plot [58], with gene genealogies divided into three internode groups and effective population size function fitted with a piecewise constant function of population size change. Both analyses were run using BEAST software for 20,000,000 MCMC iterations with a 10% burn-in and a thinning interval of 1,000. The input file for BEAST is provided as Additional File 13. As local domestication and/or introgression from aurochs into domestic breeds in Italy cannot be excluded [24,27-29], these analyses were perfomed both without the new *Bos primigenius *sequence and including it (i.e., assuming that Italian aurochs were direct ancestor of modern Italian breeds). The constant population size model and the Bayesian skyline were compared by means of Bayes Factor, computed as described above. In these analyses, the mutation rate was fixed as the median values estimated from the previous BEAST phylogenetic analyses. These values, corresponding respectively to 1, 2, and 5 million years of divergence between *B. taurus and B. bison*, are 3.3*10^-8^, 1.6*10^-8^, and 6.6*10^-9^. Population sizes were estimated assuming a generation time of 7 years [60].

## List of abbreviations

HPD: highest posterior density; mtDNA: mitochondrial DNA; MYA: millions of years Ago; MY: millons of years; TMRCA: time to most recent common ancestor

## Authors' contributions

ML, GC, ER, GCa, CV, KC and GL performed aDNA laboratory analyses. PB, provided samples and radiocarbon/stratigraphic information. SM and GB performed the statistical analyses. GCo, GDB, ER and ML performed bioinformatics analyses, GB and DC conceived the project. GB, DC, SM, ML, GL and AC wrote the paper. All authors read and approved the final manuscript.

## Supplementary Material

Additional File 1 **Table S1**. 454 sequencing results: variants in the reads mapped on bovine mitochondrial genome. Click here for file

Additional File 2 **Figure S1**. Description of sequencing results. Click here for file

Additional File 3 **Table S2**. Nucleotide misincorporations among reads. Click here for file

Additional File 4 **Figure S2**. Results of singleplex PCRs, cloning and sequencing of ambiguous positions. Click here for file

Additional File 5 **Table S3**. Amplification results of independent replications of selected fragments. Click here for file

Additional File 6 **Figure S3**. Sequence Results of independent replications of selected fragments. Click here for file

Additional File 7 **Table S4**. Location of substitutions between the *Bos primigenius *from Italy (BVA2) the and *Bos primigenius *from England (CPC98) mtDNA genome sequences. Click here for file

Additional File 8 **Table S5**. Preliminary test: aminoacids D/L ratio values for the BVA2 sample. Click here for file

Additional File 9 **Figure S4**. Preliminary test: results of amplification, cloning and sequencing of mtDNA control region fragments. Click here for file

Additional File 10 **Supplementary Materials and Methods**. Additional details regarding the samples and the molecular procedures Click here for file

Additional File 11 **Input file for PHYCAS**. Input file with the parameters used for the PHYCAS analysis. The DNA sequences are not included (see Additional File 11). Click here for file

Additional File 12 **Input file for BEAST, phylogeny**. Input file used for the phylogenetic analysis in BEAST. In this file the clock is calibrated using a 1 MY divergence between *B.taurus and B. bison*. Click here for file

Additional File 13 **Input file for BEAST, demography**. Input file used for the demographic analysis in BEAST. The mutation rate in this file is median value estimated in BEAST using the Additional File 12. Click here for file
