# Supplementary material for: The Complete Mitochondrial Genome of an 11,450-year-old Aurochsen (Bos primigenius) from Central Italy
Source: BMC Evol Biol. 2011 Jan 31;11:32. doi: 10.1186/1471-2148-11-32 (PMC3039592; doi:10.1186/1471-2148-11-32)

Figure S1. Description of sequencing results. a. Frequency distribution (% on the Y-axis) of the number of reads (the upper limit of the interval is on the X-axis) available for each nucleotide position. For example, around 15% of the nucleotide positions is covered by 1000 to 2000 reads. b. Frequency distribution (% on the Y-axis) of the frequency of the most frequent nucleotide (the upper limit of the % intervals is on the X-axis). For example, at around 85% of the positions the frequency of the most frequent allele among reads is between 99% and 100%.

a)

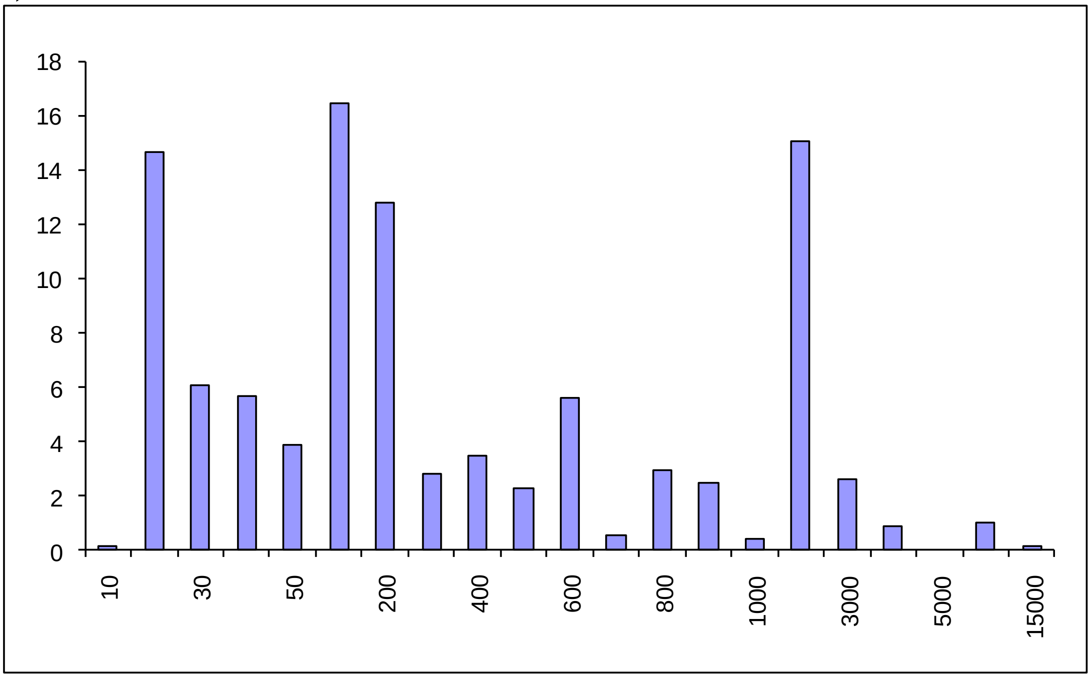

b)

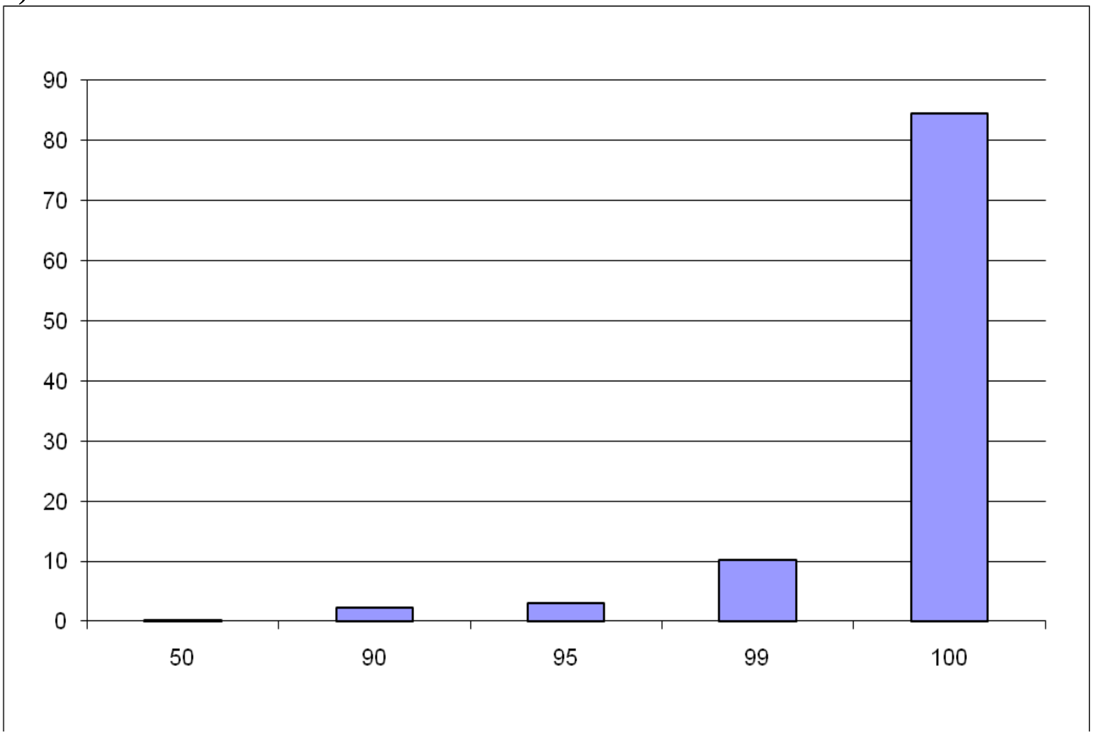

Supplement: Additional File 2 — Figure S1. Description of sequencing results. [file 1471-2148-11-32-S2.PDF]
