# Supplementary material for: The Complete Mitochondrial Genome of an 11,450-year-old Aurochsen (Bos primigenius) from Central Italy
Source: BMC Evol Biol. 2011 Jan 31;11:32. doi: 10.1186/1471-2148-11-32 (PMC3039592; doi:10.1186/1471-2148-11-32)
Supplement: Additional File 3 — Table S2. Nucleotide misincorporations among reads. [file 1471-2148-11-32-S3.DOC]

**Table S2. Nucleotide misincorporations among reads.**

The distributions of each possible misincorporation are reported (a. transitions, b. transversion). %P(TsI), %P(TsII) and %P(Tv) refer to the observed frequencies (%) of type I and type II transitions and transversion per site (ignoring indels) and corrected for nucleotide composition.

Calculation was performed on 7,565,547 nucleotide sites following Hofreiter M, Jaenicke V, Serre D, Von Haeseler A, Pääbo S (2001). DNA sequences from multiple amplifications reveal artifacts induced by cytosine deamination in ancient DNA. Nucleic Acids Res 29: 4793-4799.

| a. |  |  |  |  |  |  |
| --- | --- | --- | --- | --- | --- | --- |
| **AT>GC (TsI)** | **GC>AT (TsII)** | **Total** | **%AT** | **%GC** | **P(TsI)** | **P(TsII)** |
| 5901 | 3876 | 9777 | 60.54% | 39.46% | 0.050 | 0.079 |
|  |  |  |  |  |  |  |
| b. |  |  |  |  |  |  |
| **AT>TA** | **CG>GC** | **GC>TA** | **AT>CG** | **Total** | **P(Tv)** |  |
| 976 | 224 | 703 | 575 | 2478 | 0.033 |  |
