# Supplementary material for: The Complete Mitochondrial Genome of an 11,450-year-old Aurochsen (Bos primigenius) from Central Italy
Source: BMC Evol Biol. 2011 Jan 31;11:32. doi: 10.1186/1471-2148-11-32 (PMC3039592; doi:10.1186/1471-2148-11-32)
Supplement: Additional File 4 — Figure S2. Results of singleplex PCRs, cloning and sequencing of ambiguous positions. [file 1471-2148-11-32-S4.PDF]

Figure S2. Results of singleplex PCRs, cloning and sequencing of ambiguous positions.
Eight indels and two C->T substitutions in the BVA2 sequence generated by multiplex PCRs and pyrosequencing were checked by novel singleplex PCRs with subsequent cloning and Sanger sequencing of the amplification products. Positions are numbered according to BRS. Nucleotides identical to the reference sequence are indicated by dots.

123-250 (221+2C)

```
ref      TGTAGCTGGACTTAACTGCATCTTGAGCACCAGCATAATGATAAGCATGGACATTACAGT
CLO1     ............................................................
CLO2     ............................................................
CLO3     ............................................A...............
CLO4     ............................................................
CLO5     ..........................................................A.
         ******************************************** ************* *

                                               2
                                               2
                                               1
ref      CAATGGTCACAGGACATAAATTATATTATATATCCCCCC-TTCATAAAAATTTCCCCCTT
CLO1     ............................................................
CLO2     .......T...............................C....................
CLO3     ............................................................
CLO4     .......T....................................................
CLO5     ............................................................
         ******* ******************************* ********************

ref      AAATATCTA
CLO1     .........
CLO2     .........
CLO3     .........
CLO4     .........
CLO5     ...A.....
         *** *****
```

237-399 (362-363del)

```
ref      CCCTTAAATATCTACCACCACTTTTAACAGACTTTTCCCTAGATACTTATTTAAATTTTT
CLO1     ......................................................T.....
CLO2     ............................................................
CLO3     ............................................................
CLO4     ............................................................
CLO5     ............................................................
CLO6     .............................A..............................
         ***************************** ************************ *****

ref      CACGCTTTCAATACTCAATTTAGCACTCCAAACAAAGTCAATATATAAACGCAGGCCCCC
CLO1     ...A...................................................G....
CLO2     .........................T.............................G....
CLO3     .........................T..................................
CLO4     ............................................................
CLO5     ............................................................
CLO6     ............................................................
         *** ********************* ***************************** ****

               3
               6
               3
ref      CCCCCCCGTTGATGTAGCTTAACCCAAAGCAAGGCACTGAAAA
CLO1     ...........................................
CLO2     ...........................................
CLO3     ...........................................
CLO4     ...........................................
CLO5     ......G....................................
CLO6     .....GG....................................
         *****  ************************************
```

561-721 (587+C)

```
                                   5
                                   8
                                   7
ref      TCACGACGCCTTGCTTAACCACACCCC-ACGGGAAACAGCAGTGACAAAAATTAAGCCAT
CLO1     .T.........................C................................
CLO2     ...........................C................................
CLO3     ...........................C................................
CLO4     ...........................C....A...........................
CLO5     ...........................C................................
         * ************************* **** ***************************

ref      AAACGAAAGTTTGACTAAGTTATATTAATTAGGGTTGGTAAATCTCGTGCCAGCCACCGC
CLO1     ............................................................
CLO2     ............................................................
CLO3     ............................................................
CLO4     ............................................................
CLO5     ...........................................T................
         ******************************************* ****************

ref      GGTCATACGATTAACCCAAGCTAACAGGAGTACGGCGTAAAA
CLO1     ..........................................
CLO2     ..........................................
CLO3     ..........................................
CLO4     .....................................A....
CLO5     ..........................................
         ************************************* ****
```

1981-2161 (2048 C->T)

```
ref      AATTAAGAAAGCGTTAAAGCTCAACAACAAAAATTAAATAGATTCCAACAACAAATGAT
CLO1     ...........................................................
CLO2     ........................................................A..
CLO3     ...........................................................
CLO4     ........................................................A..
CLO5     ...........................................................
         ******************************************************** **

               2
               0
               4
               8
ref      TAACTCCTAGCCCCAATACTGGACTAATCTATTATAGAATAGAAGCAATAATGTTAATA
CLO1     .......................T...................................
CLO2     ...........................................................
CLO3     ...........................................................
CLO4     ...........................................................
CLO5     .......................T...................................
         *********************** ***********************************

ref      TGAGTAACAAGAAAAATTTTCTCCTTGCATAAGTCTAAGTCAGTGCCTGATAATACTCT
CLO1     ...........................................................
CLO2     ...........................................................
CLO3     ...........................................................
CLO4     ...........................................................
CLO5     ....................T......................................
         ******************** **************************************

ref      G
CLO1     .
CLO2     .
CLO3     .
CLO4     .
CLO5     .
         *
```

3269-3441 (3341 C->T)

```
ref     ATTAAAGAACCACTACGACCCGCTACATCTTCAGCCTCAATATTTATCCTAGCACCTAT
CLO1    ...........................................................
CLO2    ...........................................................
CLO3    ...........................................................
CLO4    .....................A.....................................
CLO5    ...........................................................
        ********************* *************************************

                     3
                     3
                     4
                     1
ref     CATAGCTTTAGGCCTAGCCTTAACCATGTGAATTCCCCTACCAATACCCTATCCTCTTA
CLO1    ...........................................................
CLO2    ...........................................................
CLO3    ...........................................................
CLO4    ...........................................................
CLO5    ....................................................T......
        **************************************************** ******

ref     TCAACATAAACCTAGGAGTCCTATTTATACTAGCCATATCAAGCCTAGCCGTATA
CLO1    ......................T...............................-
CLO2    ...........T...........................................
CLO3    .......................................................
CLO4    .......................................................
CLO5    .......................................................
        *********** ********** ******************************* 
```

8144-8308 (8258+A)

```
ref     ACGTCAACATGACTGACAATGATCTTATCAATATTCTTGACCCTTTTTATCATCTTTCA
CLO1    ...........................................................
CLO2    ...........................................................
CLO3    ...........................................................
CLO4    ...................................T.......................
CLO5    ...........................................................
        *********************************** ***********************

                                                               8
                                                               2
                                                               5
                                                               8
ref     ACTAAAAGTTTCAAAACACAACTTTTATCACAATCCAGAACTGACACCAACAAAAATAT
CLO1    .....................................A.....................
CLO2    ...........................................................
CLO3    ...........................................................
CLO4    ...........................................................
CLO5    .....................................A.....................
        ************************************* *********************

ref     TAAAACAAAACACCCCTTGAGAAACAAAATGAACGAAAATTTATTTA
CLO1    ..................................A............
CLO2    ...............................................
CLO3    ...............T...............................
CLO4    ...............................................
CLO5    ...............................................
        *************** *******************************
```

12624-12750 (12676+C)

```
                                                            1
                                                            2
                                                            6
                                                            7
                                                            6
ref     CTTATATAACCGCATCGGCGACATTGGTTTCATTTTAGCAATAGCATGGTTCCTAACAA
CLO1    ..................G........................................
```

```
CLO2     ...........................................................
CLO3     ...........................................................
CLO4     ..............................T............................
CLO5     ..............................T............................
         ****************** *********** ****************************

ref      ATCTCAATACCTGAGACCTCCAACAGATCTTCATACTAAACCCAAGCGACTCAAACATA
CLO1     ..............A............................................
CLO2     ...........................................................
CLO3     ...........................................................
CLO4     ...........................................................
CLO5     ...........................................................
         ************** ********************************************

ref      CCCTTGATT
CLO1     .........
CLO2     .........
CLO3     .........
CLO4     .....A...
CLO5     .........
         ***** ***
```

13684-13870 (13801+A)

```
ref      TTATACATCGCCTAGCTCCATACATAAATTTATCAATAAGCCAAAAATCAGCATCCTCC
CLO1     ................................................T..........
CLO2     ...........................................................
CLO3     ...........................................................
CLO4     ...........................................................
CLO5     ...........................................................
         ************************************************ **********

                                                1
                                                3
                                                8
                                                0
                                                1
ref      CTTCTAGACCTAATCTGACTAGAAGCCATCCTACCAAAAACCATCTCACTCGCCCAAAT
CLO1     ...........................................................
CLO2     .............................TT............................
CLO3     ...........................................................
CLO4     ...........................................................
CLO5     ...........................................................
         *****************************  ****************************

ref      AAAAGCATCTACCCTGGTCACAAACCAAAAAGGCCTGATCAAACTATATT
CLO1     ..................................................
CLO2     ..................................................
CLO3     ..................................................
CLO4     ..................................................
CLO5     ....................................A.............
         ************************************ *************
```
