# Supplementary material for: The Complete Mitochondrial Genome of an 11,450-year-old Aurochsen (Bos primigenius) from Central Italy
Source: BMC Evol Biol. 2011 Jan 31;11:32. doi: 10.1186/1471-2148-11-32 (PMC3039592; doi:10.1186/1471-2148-11-32)
Supplement: Additional File 5 — Table S3. Amplification results of independent replications of selected fragments. [file 1471-2148-11-32-S5.DOC]

**Table S3. Amplification results of independent replications of selected fragments.**

a. Amplification results in Trento and in Uppsala. 7 out 8 amplicons were successfully amplified in Trento or Uppsala laboratories b. Amplification results in Adelaide. 4 out 8 amplicons were successfully amplified in the Adelaide laboratory

a.

| **Positions** | **Amplification Success, Trento** | **Amplification Success, Uppsala** |
| --- | --- | --- |
| 561 -721 | yes, | no |
| 2516 -2634 | yes | not performed |
| 9580 -9743 | not performed | yes |
| 12460 -12635 | not performed | yes |
| 12624 -12750 | yes | not performed |
| 13170 -13347 | yes | no |
| 14118 -14268 | no | not performed |
| 15330 -15500 | yes | no |

b.

| **Fragment/Positions** | **Amplification Success, Adelaide** |
| --- | --- |
| CR Frag_16107-16173 | yes |
| 12S Frag_1007-1102 | yes |
| SetA_1_15771-15866 | yes |
| SetB_1_15836-16006 | no |
| SetA_2_15961-16144 | no |
| SetB_2_16042-16173 | no |
| SetA_3_16175-16304 | no |
| SetB_3_16288-059 | yes |
