# Supplementary material for: The Complete Mitochondrial Genome of an 11,450-year-old Aurochsen (Bos primigenius) from Central Italy
Source: BMC Evol Biol. 2011 Jan 31;11:32. doi: 10.1186/1471-2148-11-32 (PMC3039592; doi:10.1186/1471-2148-11-32)
Supplement: Additional File 6 — Figure S3. Sequence Results of independent replications of selected fragments. [file 1471-2148-11-32-S6.PDF]

Figure S3. Sequence Results of independent replications of selected fragments.
Comparison between BVA2 consensus sequence obtained in Florence and sequences generated in three other ancient DNA laboratories. Positions are numbered according to BRS. Nucleotides identical to the reference sequence are indicated by dots.

Uppsala:

9580-9743

```
ref                   ACGGCCTCCACGTCATCATTGGGTCCACCTTCTTAATTGTCTGCTTCTTCCGCCAATTAA
Uppsala_consensus     ............................................................
FLO_consensus         ............................................................
                      ************************************************************

                                                                9
                                                                6
                                                                8
                                                                2
ref                   AATTTCATTTTACTTCTAACCACCACTTCGGCTTTGAAGCCGGTGCCTGATACTGACATT
Uppsala_consensus     .......................................M..C.................
FLO_consensus         ..........................................C.................
                      *************************************** ** *****************

ref                   TCGTAGACGTAGTCTGACTTTTCCTCTATGTTTCTATCTATTGA
Uppsala_consensus     ............................................
FLO_consensus         ............................................
                      ********************************************
```

12460-12635

```
ref                   TTCAAATATCTACTCCTATTCCTCATTACTATGCTCATCCTTGTAACCGCAAACAACCTC
Uppsala_consensus     ............................................................
FLO_consensus         ............................................................
                      ************************************************************

ref                   TTCCAGCTATTCATTGGCTGAGAAGGCGTCGGAATCATATCATTTCTACTCATCGGATGA
Uppsala_consensus     ............................................................
FLO_consensus         ............................................................
                      ************************************************************

ref                   TGATACGGACGAGCAGATGCAAACACAGCAGCCCTACAAGCAATCTTATATAACCG
Uppsala_consensus     ........................................................
FLO_consensus         ........................................................
                      ********************************************************
```

Trento:

561-721

```
                                                5
                                                8
                                                7
ref                   TCACGACGCCTTGCTTAACCACACCCC-ACGGGAAACAGCAGTGACAAAAATTAAGCCAT
CLO1_TRENTO           ...........................C................................
CLO2_TRENTO           ...........................C......................T.........
CLO3_TRENTO           ...........................C................................
CLO4_TRENTO           ...........................C................................
CLO5_TRENTO           ...........................C................................
                      *************************** ********************** *********
FLO_consensus         ...........................C................................

ref                   AAACGAAAGTTTGACTAAGTTATATTAATTAGGGTTGGTAAATCTCGTGCCAGCCACCGC
CLO1_TRENTO           ............................................................
CLO2_TRENTO           ............................................................
```

```
CLO3_TRENTO             ............................................................
CLO4_TRENTO             ..............T.............................................
CLO5_TRENTO             ............................................................
                        ************** *********************************************
FLO_consensus           ............................................................

ref                     GGTCATACGATTAACCCAAGCTAACAGGAGTACGGCGTAAAA
CLO1_TRENTO             ..........................................
CLO2_TRENTO             .A........................................
CLO3_TRENTO             ..........................................
CLO4_TRENTO             ..........................................
CLO5_TRENTO             .A........................................
                        * ****************************************
FLO_consensus           ..........................................
```

2516-2634

```
                                            2
                                            5
                                            3
                                            6
ref                     GAAGACCCTATGGAGCTTTACCTAACCAACCCAAAGAGAATAGATTTAACCATTAAGGAA
CLO1_TRENTO             ....................A............................T..........
CLO2_TRENTO             ....................A.......................................
CLO3_TRENTO             ....................A.......................................
CLO4_TRENTO             ....................A............................T..........
CLO5_TRENTO             ....................A.......................................
                        ******************** **************************** **********
FLO_consensus           ....................A.......................................

ref                     TAACAACAATCTCCATGAGTTGGTAGTTTCGGTTGGGGTGACCTCGGAGAATAAAAAAT
CLO1_TRENTO             .........................................................-.
CLO2_TRENTO             ...........................................................
CLO3_TRENTO             ..........................................................A
CLO4_TRENTO             ...........................................................
CLO5_TRENTO             ...........................................................
                        *********************************************************
FLO_consensus           ...........................................................
```

12624-12750

```
ref                     TCTTATATAACCGCATCGGCGACATTGGTTTCATTTTAGCAATAGCATGGTTCCTAACAA
CLO1_TRENTO             ....................................................T.......
CLO2_TRENTO             ......................................A.....................
CLO3_TRENTO             ............................................................
CLO4_TRENTO             ............................................................
CLO5_TRENTO             ....................T.......................................
                        ******************** ***************** ************* *******
FLO_consensus           ............................................................

                                                                              1
                                                                              2
                                                                              7
                                                                              3
                                                                              8
ref                     ATCTCAATACCTGAGACCTCCAACAGATCTTCATACTAAACCCAAGCGACTCAAACATAC
CLO1_TRENTO             ......................................................T.....
CLO2_TRENTO             ......................................................T.....
CLO3_TRENTO             ......................................................T.....
CLO4_TRENTO             ......................................................T.....
CLO5_TRENTO             ......................................................T.....
                        ****************************************************** *****
FLO_consensus           ......................................................T.....
```

```
                       1
                       2
                       7
                       4
                       4
ref                    CCTTGAT
CLO1_TRENTO            T......
CLO2_TRENTO            T......
CLO3_TRENTO            T......
CLO4_TRENTO            T......
CLO5_TRENTO            T......
                       ******
FLO_consensus          T......
```

13170-13347

```
ref                    AGATATTCGAAAAATAGGAGGCCTATTTAAAGCCATGCCATTCACCACAACAGCCCTCAT
CLO1_TRENTO            ............................................................
CLO2_TRENTO            ................................GG..........................
CLO3_TRENTO            ............................................................
CLO4_TRENTO            ............................................................
CLO5_TRENTO            ............................................................
                       ********************************  **************************
FLO_consensus          ............................................................

ref                    TGTTGGCAGTCTCGCACTAACAGGAATACCCTTCCTCACAGGATTCTACTCCAAAGACCT
CLO1_TRENTO            ........A...................................................
CLO2_TRENTO            ..........................................G.................
CLO3_TRENTO            ............................................................
CLO4_TRENTO            ........A...................................................
CLO5_TRENTO            ............................................................
                       ******** ********************************* *****************
FLO_consensus          ............................................................

                                           1
                                           3
                                           3
                                           1
                                           0
ref                    AATCATCGAAGCCGCCAACAAGTCTTATACCAACGCCTGAGCCCTTCTAATAACATTA
CLO1_TRENTO            ....................C.....................................
CLO2_TRENTO            ....................C.....................................
CLO3_TRENTO            ....................C.....................................
CLO4_TRENTO            ................... C................C....................
CLO5_TRENTO            ....................C.....................................
                       ******************** **************** ********************
FLO_consensus          ....................C.....................................
```

15330-15500

```
                                                                             1
                                                                             5
                                                                             3
                                                                             8
                                                                             4
ref                    TACTTCTTATTTGCATACGCAATCTTACGATCAATCCCCAACAAACTAGGAGGAGTACTA
CLO1_TRENTO            ......................................................A.....
CLO2_TRENTO            ......................................................A.....
CLO3_TRENTO            .......................................C..............A.....
CLO4_TRENTO            ......................................................A.....
CLO5_TRENTO            ......................................................A.....
                       *************************************** ************** *****
FLO_consensus          ......................................................A.....
```

```
ref                    GCCCTAGCCTTCTCTATCCTAATTCTTGCTCTAATCCCCCTACTACACACCTCCAAACAA
CLO1_TRENTO            ............................................................
CLO2_TRENTO            ............................................................
CLO3_TRENTO            ............................................................
CLO4_TRENTO            ........................T...................................
CLO5_TRENTO            ........................T...................................
                       ************************ ***********************************
FLO_consensus          ............................................................

ref                    CGAAGCATAATATTCCGACCACTCAGCCAATGCCTATTCTGAGCCCTAGTA
CLO1_TRENTO            ...................................................
CLO2_TRENTO            ...................................................
CLO3_TRENTO            .........................A.........................
CLO4_TRENTO            ...................................................
CLO5_TRENTO            ...................................................
                       ************************* *************************
FLO_consensus          ...................................................
```

Adelaide

15771-15866

```
ref                    TTCTATTTAAACTATTCCCTGAACACTATTAATATAGTTCCATAAATACAAAGAGCCTTA
Adelaide_consensus     ............................................................
FLO_consensus          ............................................................
                       ************************************************************

ref                    TCAGTATTAAATTTATCAAAAATCCCAATAACTCAA
Adelaide_consensus     ....................................
FLO_consensus          ....................................
                       ************************************
```

16288-051

```
ref                    ATCTCATCTAAAACGGTCCATTCTTTCCTCTTAAATAAGACATCTCGATGGACTAATGGC
Adelaide_consensus     ............................................................
FLO_consensus          ............................................................
                       ************************************************************

ref                    TAATCAGCCCATGCTCACACATAACTGTGCTGTCATACATTTGGTATTTT
Adelaide_consensus     ..................................................
FLO_consensus          ..................................................
                       **************************************************
```

16107-16173

```
ref                    ATTCATTCTTGATAGTATATCTATTATATATTCCTTACCATTAGATCACGAGCTTAATTA
Adelaide_consensus     ...............Y............................................
FLO_consensus          ............................................................
                       *************** ********************************************

ref                    ACCATGCC
Adelaide_consensus     ........
FLO_consensus          ........
                       ********
```

1007-1102

```
ref                    AACCTCACCAATTCTTGCTAATACAGTCTATATACCGCCATCTTCAGCAAACCCTAAAAA
Adelaide_consensus     ............................................................
FLO_consensus          ............................................................
                       ************************************************************

ref                    GGAAAAAAAGTAAGCGTAATTATGATACATAAAAAC
Adelaide_consensus     ....................................
FLO_consensus          ....................................
```

****
