# Supplementary material for: The Complete Mitochondrial Genome of an 11,450-year-old Aurochsen (Bos primigenius) from Central Italy
Source: BMC Evol Biol. 2011 Jan 31;11:32. doi: 10.1186/1471-2148-11-32 (PMC3039592; doi:10.1186/1471-2148-11-32)
Supplement: Additional File 7 — Table S4. Location of substitutions between the Bos primigenius from Italy (BVA2) the and Bos primigenius from England (CPC98) mtDNA genome sequences. [file 1471-2148-11-32-S7.DOC]

**Table S4. Location of substitutions between the *Bos primigenius* from Italy (BVA2) the and *Bos primigenius* from England (CPC98) mtDNA genome sequences.**

a. substitutions located in the control region. b. substitutions located in genes for rRNA (12S, 16S) and tRNA (M = tRNA-Met; N = tRNA-Asn; D = tRNA-Asp; S = tRNA-Ser; T = tRNA-Thr). c. substitutions located in protein genes(ND1-6 = NADH dehydrogenase subunits 1-6; COX1,2 = cytochrome c oxidase subunits 1 and 2; ATP8,6 = ATP synthase subunits 8 and 6; CYTB = cytochrome b)

a.

|  | 15951 | 15953 | 15994 | 16019 | 16049 | 16051 | 16058 | 16074 | 16085 | 16121 | 16122 | 16141 | 16231 | 16255 | 16264 | 16301 | 190 | 222 | 249 | 300 | 301 | 363 |
| --- | --- | --- | --- | --- | --- | --- | --- | --- | --- | --- | --- | --- | --- | --- | --- | --- | --- | --- | --- | --- | --- | --- |
| BVA2 | T | C | A | T | C | T | C | T | T | G | T | T | C | T | G | C | C | T | T | G | C | C |
| CPC98 | C | G | G | C | T | C | T | C | C | R | C | C | T | C | A | T | T | C | C | A | T | - |

b.

|  | 587+C | 588+C | 1128 | 1481 | 2145 | 2558 | 2585 | 4252 | 5501 | 7356 | 12016 | 15673 |
| --- | --- | --- | --- | --- | --- | --- | --- | --- | --- | --- | --- | --- |
| BVA2 | C | - | A | G | G | G | T | T | C | A | T | T |
| CPC98 | - | C | G | A | A | A | C | C | T | G | C | C |
|  | 12s | 12s | 12s | 16s | 16s | 16s | 16s | M | N | D | S | T |

c.

|  | 3379 | 3550 | 4293 | 4676 | 5156 | 5743 | 5890 | 5899 | 6160 | 6436 | 7952 | 7994 | 8236 | 8358 | 8370 | 10126 | 10691 | 11000 | 11140 | 11740 | 12377 | 12433 |
| --- | --- | --- | --- | --- | --- | --- | --- | --- | --- | --- | --- | --- | --- | --- | --- | --- | --- | --- | --- | --- | --- | --- |
| BVA2 | T | G | C | A | G | T | C | A | T | G | C | A | T | C | A | C | C | C | A | C | T | T |
| CPC98 | C | A | T | G | A | C | T | G | C | A | T | G | C | T | C | T | G | T | G | T | C | C |
|  | ND1 | ND1 | ND2 | ND2 | ND2 | COX1 | COX1 | COX1 | COX1 | COX1 | COX2 | COX2 | ATP8 | ATP6 | ATP6 | ND3 | ND4 | ND4 | ND4 | ND4 | ND5 | ND5 |

|  | 12468 | 12469 | 12525 | 12675 | 12738 | 12744 | 12750 | 13005 | 13821 | 14036 | 14129 | 14159 | 14580 | 14873 | 15134 | 15384 | 15627 |
| --- | --- | --- | --- | --- | --- | --- | --- | --- | --- | --- | --- | --- | --- | --- | --- | --- | --- |
| BVA2 | T | C | G | C | T | T | T | A | A | G | G | G | G | G | C | A | A |
| CPC98 | C | T | A | T | C | C | C | G | G | A | T | A | A | A | T | G | G |
|  | ND5 | ND5 | ND5 | ND5 | ND5 | ND5 | ND5 | ND5 | ND5 | ND6 | ND6 | ND6 | CYTB | CYTB | CYTB | CYTB | CYTB |
