# Supplementary material for: The Complete Mitochondrial Genome of an 11,450-year-old Aurochsen (Bos primigenius) from Central Italy
Source: BMC Evol Biol. 2011 Jan 31;11:32. doi: 10.1186/1471-2148-11-32 (PMC3039592; doi:10.1186/1471-2148-11-32)
Supplement: Additional File 8 — Table S5. Preliminary test: aminoacids D/L ratio values for the BVA2 sample. [file 1471-2148-11-32-S8.DOC]

**Table S5. Preliminary test: aminoacids D/L ratio values for the BVA2 sample.**

D/L ratio values of alanine, aspartic acid and glutamine determined in three different injections starting from 3.03mg of sample (according to protocol described in Poinar et al. 1996)

| **Injection** | **D/L Alanine** | **D/L Aspartic A.** | **D/L Glutamine** |
| --- | --- | --- | --- |
| **I** | 0,019728 | 0,088908 | 0,043761 |
| **II** | 0,025643 | 0,032049 | 0,014808 |
| **III** | 0,017785 | 0,090376 | 0,032806 |
| **Average** | **0,021** | **0,070** | **0,030** |
| **Stand. Dev.** | **0,004** | **0,033** | **0,015** |
