# Supplementary material for: The Complete Mitochondrial Genome of an 11,450-year-old Aurochsen (Bos primigenius) from Central Italy
Source: BMC Evol Biol. 2011 Jan 31;11:32. doi: 10.1186/1471-2148-11-32 (PMC3039592; doi:10.1186/1471-2148-11-32)

Figure S4. Preliminary test: results of amplification, cloning and sequencing of mtDNA control region fragments.

The first lines report the reference sequence (BRS, Anderson et al. 1982) with the numbering of the nucleotide positions. Nucleotides identical to the reference sequence are indicated by dots. In the first column each clone is identified by extraction number and PCR number. Primer pair used are: L16030 5’ACATTAAATTATATGCCCCATGC3’and H16165 5’TTCACGCGGCATGGTA3’; L16159 5’TTCCTTACCATTAGATCACGAGC 3’ and H16276 5’GATGAGATGGCCCTGAAGAA3’; L16267 5’CAATGAATTTTACCAGGCAT3’ and H00034 5’CCAAATGTGACAGCACAG3’.

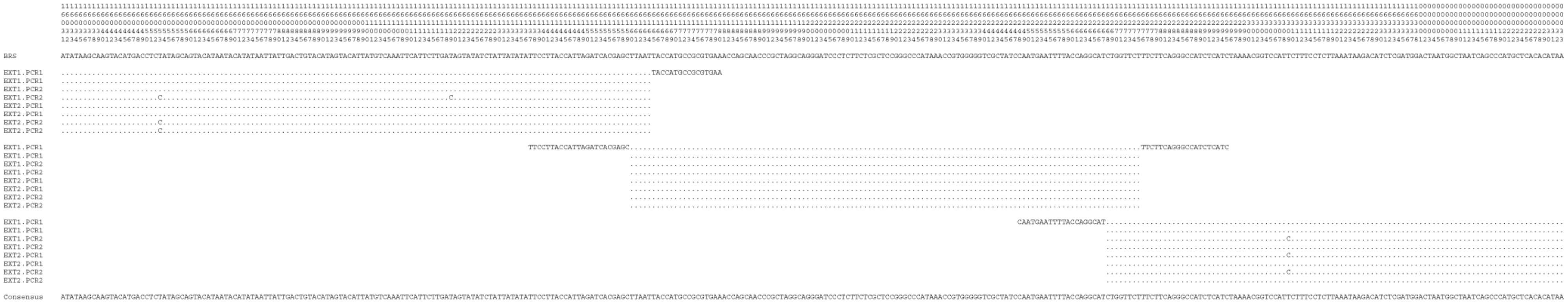

Supplement: Additional File 9 — Figure S4. Preliminary test: results of amplification, cloning and sequencing of mtDNA control region fragments. [file 1471-2148-11-32-S9.PDF]
