# Supplementary material for: The Complete Mitochondrial Genome of an 11,450-year-old Aurochsen (Bos primigenius) from Central Italy
Source: BMC Evol Biol. 2011 Jan 31;11:32. doi: 10.1186/1471-2148-11-32 (PMC3039592; doi:10.1186/1471-2148-11-32)
Supplement: Additional File 10 — Supplementary Materials and Methods. Additional details regarding the samples and the molecular procedures [file 1471-2148-11-32-S10.DOC]

**Additional details regarding the sample and the molecular procedures**

**General equipment**

DNA was extracted in four laboratories (Florence, Trento, Uppsala and Adelaide) dedicated exclusively to ancient DNA work. All DNA extractions and PCR set ups were carried out in labs physically separated from those in which PCR cycling and post-PCR analysis was conducted. Multiple measures were undertaken to exclude contamination and potential artifactual DNA changes. Disposable masks, gloves, and laboratory coats were worn throughout and were changed frequently; pipettors were UV-irradiated in between use. All DNA extractions and PCR reactions included multiple negative controls.

**The sample**

We selected for the analysis a bone fragment (phalanx) named BVA2 from the Vado all’Arancio site. Radiocarbon dating on two associated samples resulted in an age of 11.300±150 years BP (R1333, analysis performed in Rome) and 11,600±130 years BP (Ly 3415, analysis performed in Lyon). About 7g of the bone was broken in 4 pieces, about 1.5-2.0g each. Two bone pieces were analysed in the Florence laboratory, and the remaining two bone fragments were analysed in the Trento laboratory and in the Adelaide facility. An aliquot of bone powder from one of the Florence fragments was sent to Uppsala.

**DNA extraction**

In the Florence lab, all DNA-preparation and extraction methods adhered to ancient DNA requirements (Cooper and Poinar 2000; Paabo et al. 2004; Willerslev and Cooper 2005). To prevent contamination prior handling, the outer layer of bone fragments was removed using a rotary tool. The fragments were briefly soaked in 10% bleach and then irradiated for 1 hour under UV light. After decontamination, each bone fragment was powdered and DNA was extracted independently from the two powder aliquots using a silica-based protocol (modified from Caramelli et al. 2003). Multiple negative controls were included in each extraction.

**Quantification of DNA Molecules**

Real-time PCR amplification was performed using Brilliant® SYBR® Green QPCR Master Mix (Stratagene) in MX3000P (Stratagene) in a total volume of 50μl with 0.5μM of a reverse primer located at 16030 and forward primer located at H16165. Five microliters of a 1:50 dilutions of each extract were amplified. Thermal cycling conditions were 95°C for 10 min, 40 cycles at 95°C for 30 s, 53°C for 1 min and 72°C for 30 s, followed by SYBR® Green dissociation curve step. Appropriate ten-fold serial dilutions of the purified and quantified standard were included in the experiment to create the standard curve in order to quantify the number of initial DNA molecules in the samples. Both unknown samples and standard’s serial dilutions were amplified in duplicate.

**Multiplex PCR amplification**

Multiplex PCR was performed in a final volume of 20 µl containing 5 µl of aDNA extract (diluted 1:50 to reduce inhibitor effects), 1X PCR buffer, 4mM MgCl2, 0.15 µM of each primer, 250 µM of each dNTP, and 2 U of AmpliTaq Gold (Applied Biosystems). Multiplex PCR conditions began with an initial denaturation at 94 °C for 9 min, followed by 27 cycles of 94 °C for 20 sec, 50 °C for 30 sec and 72 °C for 30 sec. Multiplex PCR products were then diluted to 1:10 and used as a template for a second round of individual simplex PCRs, where only one primer pair was used. The simplex PCRs were performed in a final volume of 20 µl containing 5 µl of diluted multiplex PCR product, 1X PCR buffer, 4 mM MgCl2, 1 µM of each primer, 250 µM of each dNTP and 2 U AmpliTaq Gold (Applied Biosystems). The simplex PCR conditions were the same of the multiplex PCRs except for cycles numbers that were 33 instead of 27 and annealing temperature that were raised to 52°C.

Each multiplex PCR reaction was accompanied by two contamination controls: an extraction blank and a PCR negative control. After an intial amplification, both controls were diluted 1:10 and re-amplified in each secondary singleplex PCR. A supplemental negative control was introduced in each singleplex All contamination controls were checked by electrophoresis on 2% agaorse gel with a 100bp DNA ladder and, if amplification products of the expected sizes of the amplicons were detected, PCR was discarded and repeated.

Full details about primers used for multiplex PCRs (position respect to BRS, sequence, amplicon length) are listed in the table below.

Set A:

| **Forward** | | **Sequence (5'->3')** | **Reverse** | | **Sequence (5'->3')** | **Lenght (bp)** |
| --- | --- | --- | --- | --- | --- | --- |
| L | 22-43 | GCTCACACATAACTGTGCTGTC | H | 178-198 | TGTCCTGTGACCATTGACTGT | 177 |
| L | 541-560 | TCAAGCACACACCCTGTAGC | H | 722-743 | TTTGGTATGGTGCTTTAACACG | 203 |
| L | 925-947 | GCAACAGCTTAAAACTCAAAGGA | H | 1118-1140 | CATTTCTTCCCATTTCATAGGTT | 216 |
| L | 1536-1559 | GGAGATAGAAATCTAAGTACGGCG | H | 1696-1715 | AGTAGCTCGTCTGGTTTCGG | 180 |
| L | 1963-1982 | TAGGCCTAAAAGCAGCCATC | H | 2162-2188 | TGGATTATTTTTATTGACTGTTAGTGG | 226 |
| L | 2495-2515 | GGGAATGCACAAATAAGACGA | H | 2635-2654 | TCTTTAAAATCGCTCGGAGG | 160 |
| L | 3005-3024 | TGCCCTAGAACAGGGCTTAG | H | 3154-3176 | TTCGTTCCACTAACGTAAGGAAT | 172 |
| L | 3450-3469 | TCTGATCAGGCTGAGCTTCC | H | 3630-3649 | TATTGCTAGAGGCCATGCTG | 200 |
| L | 3971-3997 | AAAAATTTTCTACCTCTGACACTAGCC | H | 4167-4186 | GGTGTAATTGGGAGCACGAA | 216 |
| L | 4506-4525 | TCAGGCCAATGAACCGTAAT | H | 4727-4753 | AGGATTGATAAAACTGATAGGGTTAGA | 228 |
| L | 4936-4958 | CCACCACTACCCTGTCATTATC | H | 5087-5108 | GAAAGTGGGTAGAATGATGCTG | 173 |
| L | 5403-5423 | CAATTGAATGCAAATCAACCA | H | 5604-5622 | TACCAAGCCCTGTGGTGAA | 220 |
| L | 5947-5966 | TGGTGCTCCCGATATAGCAT | H | 6137-6158 | TTGAGGAAACTCCTGCTAAGTG | 212 |
| L | 6391-6416 | CTGATTCTTTGGACACCCCGAAGTCT | H | 6532-6558 | TGGTGGGCTCATACGATGAAACCTAGA | 168 |
| L | 6869-6891 | CATTGATTCCCACTATTCTCAGG | H | 7087-7111 | TGCTTCTCAGATGATGAAAACTATT | 230 |
| L | 7400-7411 | CCAAGATGCAACATCACCAA | H | 7595-7619 | TCGTAAAGAAGGAAGAGCAATTAGA | 220 |
| L | 7922-7941 | CCTTATATCGTCCCGTCCAG | H | 8114-8133 | GGCATGTCACCAAGGAGAGT | 212 |
| L | 8449-8474 | TCAAAACAAATAATGAGTATCCACAA | H | 8656-8675 | AAGAAATGGGCAAGTGATGC | 227 |
| L | 9004-9022 | ACCCAAGCCCTTGACCTCT | H | 9198-9217 | ATTCCATAACGGAGGCCTTT | 214 |
| L | 9560-9579 | TTTTGTAGCCACAGGCTTCC | H | 9744-9770 | GTTAATACTAAAAGAATAGGAGCCTCA | 211 |
| L | 10082-10102 | TGCTTACCATAGCCCTCTTCC | H | 10269-10292 | TCCTACAAGAGATACTGTGAATGC | 211 |
| L | 10449-10471 | GCAGCCCTAGGTCTATCTCTACT | H | 10648-10667 | CGCCAAACTGGTTTATGAGG | 219 |
| L | 10957-10976 | AAACGCCGGACTCTATTTCC | H | 11180-11199 | GGGGCTTCTACGTGAGCTTT | 229 |
| L | 11536-11555 | AATCCACAGCCGAACCATAA | H | 11728-11750 | GAGAATATAGGGCGGTGATTACT | 215 |
| L | 12055-12077 | GTTTATCCGTTGGTCTTAGGAA | H | 12272-12292 | TTAGTTCTTGGCCTGAGTGGA | 228 |
| L | 12604-12623 | ACAGCAGCCCTACAAGCAAT | H | 12751-12771 | GGTTGCAGCTAATGCTAGTCC | 168 |
| L | 13091-13110 | ACATCTGTACCCACGCCTTT | H | 13233-13252 | CTGTTAGTGCGAGACTGCCA | 162 |
| L | 13513-13534 | TCCAACAATATTCCTCCAACAA | H | 13707-13726 | TGTATGGAGCTAGGCGATGT | 214 |
| L | 14093-14117 | CACAACCTCAACTTCTTTATCCTTT | H | 14269-14288 | TTTGGGGTTGATGGTCTTTT | 196 |
| L | 14605-14624 | GAAATTTCGGTTCCCTCCTG | H | 14798-14817 | AGCCTCGTCCTACGTGCATA | 213 |
| L | 15126-15145 | TCCAACAACCCAACAGGAAT | H | 15314-15335 | GAAGTATCACTCGGGTTTGATG | 210 |
| L | 15511-15530 | TGACACTCACATGAATTGGA | H | 15704-15728 | TGAGTCTTAGGGAGGTTAGTTGTTC | 219 |
| L | 16096-16122 | CATTATGTCAAATTCATTCTTGATAGT | H | 16270-16289 | ATGGCCCTGAAGAAAGAACC | 194 |

Set B:

| **Forward** | | **Sequence (5'->3')** | **Reverse** | | **Sequence (5'->3')** | **Lenght (bp)** |
| --- | --- | --- | --- | --- | --- | --- |
| L | 103-122 | CCCTGACCCGGAGCATCTAT | H | 251-273 | GAAAAGTCTGTTAAAAGTGGTGG | 172 |
| L | 641-666 | ATATTAATTAGGGTTGGTAAATCTCG | H | 784-810 | GGTCACTTTCGTCATTTATTTTTATTT | 170 |
| L | 1093-1116 | ACATAAAAACGTTAGGTCAAGGTG | H | 1298-1320 | TGCGTTTAAATAGGGTTAGATGC | 228 |
| L | 1621-1643 | AGATTACCCCTTGTACCTTTTGC | H | 1801-1820 | TGGACAACCAGCTATCACCA | 200 |
| L | 2125-2147 | TTGCATAAGTCTAAGTCAGTGCC | H | 2286-2304 | TGTTTTTGGTAAACAGGCGG | 180 |
| L | 2530-2552 | GCTTTACCTAACCAACCCAAAGA | H | 2724-2743 | GGATTGCGCTGTTATCCCTA | 214 |
| L | 3072-3094 | CCAGAGATTCAAATCCTCTCCTT | H | 3291-3273 | GCGGGTCGTAGTGGTTCTT | 220 |
| L | 3608-3629 | GAACAAATATGGTTAATCCTCC | H | 3807-3832 | TCCTAGGAATAAAATTGCTGTAAAGA | 225 |
| L | 4143-4166 | CCTACTCCTAAGAATCCAAAACTC | H | 4348-4367 | TTCAAACCCGATTCAGACAA | 225 |
| L | 4699-4723 | TCTTCCCATCAATTAACCTAAACTT | H | 4891-4917 | TGGTAAATATGGTGGAAGTTATAATGA | 219 |
| L | 5055-5081 | TGAATAATCATCCAAGAGATAACAAAA | H | 5230-5256 | CGACTATTGTTGGTAGAAAAGTTATTT | 202 |
| L | 5568-5588 | AAGCTGCTTCTCTGAATTTGC | H | 5783-5802 | GCGCGAATTAGAAGGCTTAG | 229 |
| L | 6067-6086 | CGTGTACCCTCCCTTAGCAG | H | 6221-6240 | ACGAACAGAGGGGTTTGGTA | 174 |
| L | 6507-6527 | TTTGGGCTATAATGTCAATCG | H | 6700-6720 | ATAAAGCCTAGGGCTCACATT | 214 |
| L | 7047-7069 | CATCAATAGGCTCATTCATTTCC | H | 7237-7256 | GTAGGGGGTTCGATTCCTTC | 210 |
| L | 7575-7594 | CTGCCCGCCATCATCTTAAT | H | 7756-7774 | CGTAGCTCCCCTGGCTTTA | 200 |
| L | 8042-8068 | TGCGTCAATATTATAAAATCACTAAGA | H | 8193-8218 | TTTTGAAACTTTTAGTTGAAAGATGA | 177 |
| L | 8584-8604 | TCAATAAACCTAGGCATAGCCA | H | 8739-8759 | GCGAGGGCTATAGGTTGAATA | 176 |
| L | 9165-9184 | ACCTTCCAAGGGCACCATAC | H | 9383-9404 | GGTAATAGAAACTCCGGAAGCC | 230 |
| L | 9700-9723 | TCGTAGACGTAGTCTGACTTTTCC | H | 9848-9869 | AGGGTGGCTAGTGTAAAATTGG | 170 |
| L | 10150-10172 | GGACTAGAATGAACCGAATATGG | H | 10304-10324 | GAGGATATTAGGTGGGATCGG | 175 |
| L | 10624-10647 | TCTAATTAGCTTTACAAGCCTCCT | H | 10821-10844 | GGAATAGTTGTAGTGAGATCAGCA | 221 |
| L | 11155-11174 | ATATGGCCTCCACCTTTGAC | H | 11378-11399 | CAGAGGAGTATGCGATGAGTGA | 230 |
| L | 11677-11701 | GTCAACCTTTTCATGATCTAACATT | H | 11875-11898 | GGTCCTAGAATAATTTTTGGGTTT | 222 |
| L | 12242-12262 | TTACCAGCATAATTCCCACAA | H | 12436-12459 | GAATTTGTTAATATTGGGGTCTGA | 218 |
| L | 12691-12716 | ACCTGAGACCTCCAACAGATCTTCAT | H | 12827-12845 | AGTGCTGAGACGGGAGTTG | 155 |
| L | 13150-13169 | CACAGCCTAAACGACGAACA | H | 13348-13367 | GCTGTGAAAGAGGTGGCAAT | 218 |
| L | 13683-13702 | GCTAGGGTATTTCCCCACAA | H | 13871-13894 | GGATTGTGATTAAGAAGGAGAGGA | 212 |
| L | 14238-14261 | AAAACTACCATCATACCTCCCAAA | H | 14448-14473 | TCATTAGTCATGGTTAGATTCCATGT | 230 |
| L | 14763-14782 | GCAAACGGAGCTTCAATGTT | H | 14963-14982 | CGATGTATGGGATTGCTGAT | 220 |
| L | 15242-15260 | ACTATTCGCACCCGACCTC | H | 15379-15401 | GAAGGCTAGGGCTAGTACTCCTC | 160 |
| L | 15681-15703 | TGGTCTTGTAAACCAGAGAAGGA | H | 15870-15890 | TTTGGTTAGGGTGCAAATTCT | 210 |
| L | 16241-16262 | CGCTATCCAATGAATTTTACCA | H | 81-100 | TTTGACGGCCATAGCTGAGT | 198 |

Set C:

| **Forward** | | **Sequence (5'->3')** | **Reverse** | | **Sequence (5'->3')** | **Lenght (bp)** |
| --- | --- | --- | --- | --- | --- | --- |
| L | 217-236 | CCCCCTTCATAAAAATTTCC | H | 400-419 | TTGGGAGACTCATCTAGGCA | 202 |
| L | 728-748 | AAAGCACCATACCAAATAGGG | H | 925-947 | TCCTTTGAGTTTTAAGCTGTTGC | 220 |
| L | 1271-1290 | CCGTCACCCTCCTCAAATAG | H | 1461-1481 | CGAGAGGGTATCTTTGGGCTA | 211 |
| L | 1775-1797 | AGTAGAGGTGACATGCCTAACGA | H | 1919-1944 | TTACTCTCTAGTCAAGGTTGTATCCG | 170 |
| L | 2188-2162 | CCACTAACAGTCAATAAAAATAATCCA | H | 2348-2366 | CGGCCGTTAAACAGTTGTC | 205 |
| L | 2703-2723 | GATCAACGGAACAAGTTACCC | H | 2909-2929 | TTGTCCTTTCGTACTGGGAGA | 227 |
| L | 3249-3268 | CCGATGCAATCAAACTTTTC | H | 3442-3462 | CAGCCTGATCAGAGAATGGAG | 214 |
| L | 3755-3774 | CCATTTGCCCTCTTCTTCAT | H | 3906-3925 | TGCTCGGATTCATAGGAAGG | 171 |
| L | 4253-4275 | CCTTCCCGTACTAATAAACCCAA | H | 4407-4426 | TCTGTAGCTCGTGGGTTGTG | 174 |
| L | 4827-4850 | GGCTGAATAACAGCAGTACTACCA | H | 4985-5004 | TGGCAAGAATTAGGACGGTT | 178 |
| L | 5164-5186 | CCACACTAACAATATTTCCCTCC | H | 5316-5338 | AAGGCTCTTGGTCTGTTTAACCT | 175 |
| L | 5761-5781 | GGCCGGTATAGTAGGAACAGC | H | 5947-5966 | ATGCTATATCGGGAGCACCA | 206 |
| L | 6137-6158 | CACTTAGCAGGAGTTTCCTCAA | H | 6333-6352 | CGGGTCGAAGAAGGTTGTAT | 216 |
| L | 6674-6697 | GGTAATATCAAATGGTCTCCTGCT | H | 6860-6880 | TGGGAATCAATGAACAAATCC | 207 |
| L | 7208-7234 | CCCACCTATGTTAACCTAAAATAAGAA | H | 7407-7429 | TCTTCTATGATTGGTGATGTTGC | 222 |
| L | 7715-7737 | GGACTTAAGCTTCGACTCCTACA | H | 7863-7884 | TTAGTCCTAGAGAGGGCACAGC | 170 |
| L | 8124-8143 | GTGACATGCCGCAACTAGAC | H | 8309-8333 | AAAATTACAGGGGTAATAAAAGAGG | 210 |
| L | 8656-8675 | GCATCACTTGCCCATTTCTT | H | 8849-8872 | TGGTGAATGTAATTAGAGCTGTTG | 217 |
| L | 9361-9382 | TGCTCAACACCTCTGTCCTATT | H | 9560-9579 | GGAAGCCTGTGGCTACAAAA | 216 |
| L | 9740-9761 | TTGATGAGGCTCCTATTCTTTT | H | 9938-9957 | TCCTATGGGGTCAAATCCAC | 218 |
| L | 10265-10291 | AATAGCATTCACAGTATCTCTTGTAGG | H | 10444-10469 | TAGAGATAGACCTAGGGCTGCTTCAC | 205 |
| L | 10746-10772 | TCCCTCTAATACTAATAGCTAGCCAAC | H | 10898-10919 | TGAGTGTTGGGACTAGTGTTGC | 174 |
| L | 11347-11365 | CTCAATCTGCCTCCGTCAA | H | 11539-11558 | GAATTATGGTTCGGCTGTGG | 212 |
| L | 11820-11839 | CACGGGAAAATGCACTCATA | H | 11970-11993 | TTCTTGCATACTTTTTCGGTAAAT | 174 |
| L | 12377-12397 | TCCCAGTAGCACTATTCGTCA | H | 12520-12539 | CAGCCAATGAATAGCTGGAA | 163 |
| L | 12796-12814 | CACCCGTGACTTCCCTCTG | H | 12984-13006 | TTTTAATGTCATTTTGGGTGAGG | 211 |
| L | 13323-13342 | CGCCTGAGCCCTTCTAATAA | H | 13523-13542 | GGGAATTGTTGTTGGAGGAA | 220 |
| L | 13844-13863 | ACCAAAAAGGCCTGATCAAA | H | 14011-14030 | TGAAGAGGCCATAGGGATTG | 187 |
| L | 14401-14427 | CGAAAATAACGCTTAGAATAAATACAA | H | 14601-14620 | AGGGAACCGAAATTTCATCA | 220 |
| L | 14886-14907 | GCCACAGCATTTATAGGATACG | H | 15029-15048 | AGAATCGGGTAAGGGTTGCT | 163 |
| L | 15310-15329 | CTCACATCAAACCCGAGTGA | H | 15501-15523 | CATGTGAGTGTCAGTAGGTCTGC | 214 |
| L | 15847-15869 | CAAAAATCCCAATAACTCAACAC | H | 16036-16058 | GCTATAGAGGTCATGTACTTGCT | 212 |

Set D.

| **Forward** | | **Sequence (5'->3')** | **Reverse** | | **Sequence (5'->3')** | **Lenght (bp)** |
| --- | --- | --- | --- | --- | --- | --- |
| L | 375-394 | TTAACCCAAAGCAAGGCACT | H | 565-584 | GTGTGGTTAAGCAAGGCGTC | 210 |
| L | 832-851 | AGCTAAGACCCAAACTGGGA | H | 996-1015 | GGTGAGGTTTATCGGGGTTT | 184 |
| L | 1435-1459 | TCATTATGAATATCTTGAACTAGAC | H | 1629-1651 | TTCATTATGCAAAAGGTACAAGG | 217 |
| L | 1801-1820 | TGGTGATAGCTGGTTGTCCA | H | 1987-2009 | TTGTTGAGCTTTAACGCTTTCTT | 209 |
| L | 2324-2342 | TATTGGAGGCATTGCCTGC | H | 2526-2547 | GGGTTGGTTAGTTAAAGCTCCA | 224 |
| L | 2884-2907 | GGTCGGTTTCTATCTATTACGTAT | H | 3085-3107 | TGAACATTTTGTTAAGGAGAGGA | 224 |
| L | 3361-3381 | TCCCCTACCAATACCCTATCC | H | 3508-3530 | CTTCGTATGAGATTGTTTGTGCT | 170 |
| L | 3829-3848 | AGGAACATCCCACAATCCAC | H | 3998-4018 | GGATACGTGTCACATGCACAG | 190 |
| L | 4348-4367 | TTGTCTGAATCGGGTTTGAA | H | 4538-4556 | AAGTATTGAGGCTATTGGG | 209 |
| L | 4891-4917 | TCATTATAACTTCCACCATATTTACCA | H | 5049-5071 | TCTTGGATGATTATTCATTTTGG | 181 |
| L | 5243-5268 | ACCAACAATAGTCGTATTATCTACCA | H | 5439-5459 | GCCCACCAGTCTAGTGAGGAT | 217 |
| L | 5848-5868 | CAACGTAGTTGTAACCGCACA | H | 5995-6013 | GAATGAGGGAGGGAGGAGTC | 166 |
| L | 6309-6329 | TGCTATTAACAGACCGGAACC | H | 6507-6528 | CCGATTGACATTATAGCCCAAA | 220 |
| L | 6801-6826 | ACGTTGTCGCACATTTCCACTATGTT | H | 6943-6969 | TGTGGGAAGAAGGTTATATTGACGCCT | 169 |
| L | 7291-7315 | CTCTCTCAATAAACGAGGTGTTAGT | H | 7437-7456 | AGCGTGTGGTCATGAAAGTG | 166 |
| L | 7760-7779 | GCCAGGGGAGCTACGACTAT | H | 7955-7974 | CGCAAATTTCTGAGCATTGA | 215 |
| L | 8272-8292 | CACCCCTTGAGAAACAAAATG | H | 8475-8500 | TTAATGTTCATGTTTGTCCTTTAGAA | 229 |
| L | 8822-8846 | CACTTGCACTAATAAGCATTAGCA | H | 9026-9045 | AGAGGGCAGACAAAGCTCCT | 224 |
| L | 9451-9473 | AAGCCCTATTTATCACCATCACA | H | 9600-9621 | AGACAATTAAGAAGGTGGACCC | 171 |
| L | 9915-9934 | TGAGAAAACAAGCCCATACG | H | 10109-10130 | GCTAGGCTTACAGCTAGGAGGA | 216 |
| L | 10382-10405 | CCTCAACTCACATTTTACATTAGC | H | 10525-10550 | TTGGAATAATGTATTTTAGCATTGGA | 169 |
| L | 10821-10847 | TGCTGATCTCACTACAACTATTCCTAA | H | 10999-11021 | TTAGTGCGACTAATAGGGGTAGG | 201 |
| L | 11461-11479 | AGCAACCGCCCTTATGATT | H | 11620-11641 | GGGGGTAGAGCTAAGTTGGTT | 180 |
| L | 11865-11885 | TCCTAACCCTAAACCCAAAAA | H | 12082-12101 | ATTTGGAGTTGCACCAATTT | 230 |
| L | 12436-12459 | TCAGACCCCAATATTAACAAATTC | H | 12636-12653 | AAACCAATGTCGCCGATG | 218 |
| L | 12962-12983 | CACTATTTACAGCAATATGCGC | H | 13159-13181 | TTTCGAATATCTTGTTCGTCGTT | 220 |
| L | 13446-13465 | CAACCCCCTTCTGATCAACT | H | 13597-13617 | TTCTAGGGCTAAGATGAAGCC | 172 |
| L | 13959-13989 | CACCCAGTTACAATAACTAATCAGG | H | 14137-14157 | CTGGCTTGTTGATGGAGTTCT | 193 |
| L | 14526-14545 | CGAAAGTCCCACCCACTAAT | H | 14679-14698 | GCTGTTGTTGTGTCGGATGT | 173 |
| L | 14969-14988 | AATCCCATACATCGGCACAA | H | 15159-15178 | GGGTGGAATGGGATTTTGTC | 210 |
| L | 15423-15443 | ATCCCCCTACTACACACCTCC | H | 15562-15582 | GGACAGATGCTAGTTGTCCGA | 160 |
| L | 16007-16030 | TACATTAAATTATATGCCCCATGC | H | 16178-16196 | CTAGCGGGTTGCTGGTTTC | 190 |

**454 sample preparation and pyrosequencing**

In the Milan ITB-CNR laboratory, PCR products were checked by miniaturized capillary gel electrophoresis (Agilent 2100 Bioanalyzer, Agilent, Santa Clara, CA, USA), purified (MinElute PCR Purification Kit, QIAGEN, Hilden, Germany) and mixed in equimolar proportion. Due to the amplicon sizes, amplified DNA samples were not fragmented and were processed in order to obtain the single-stranded template DNA (sstDNA) library as in Roche GS FLX library preparation protocol. The quality and quantity of sstDNA was checked by Agilent Bioanalyzer and RiboGreen RNA Quantitation Kit (Invitrogen, Carlsbad, CA). sstDNA libraries were bound onto DNA capture bead and amplified by emulsion PCR (emPCR) as reported in Roche GS FLX protocol. Positive DNA beads were prepared as in Roche GS FLX protocol, counted (Multisizer 3 Coulter Counter; Beckman Coulter, Fullerton, CA, USA) and subsequently sequenced by FLX Genome Sequencer (FLX Roche/454 LifeSciences). After identifying those reads showing the PCR primers at their termini, we masked for each read the couple of primers and mapped the resulting portion on the reference sequence [NCBI accession: V00654]. This was done by the Amplicon Variant Analyzer application (AVA) by Roche, using default parameters. Finally, starting from the AVA multi-alignments, we generated the consensus sequences with a home-made Python script, which assigns for each position the most frequently present base.

**Singleplex amplification**

2μl of extracted DNA were amplified with the following protocol: 94° C for 10 min (Taq polymerase activation), followed by 50 cycles of PCR (denaturation, 94°C for 45 sec, annealing, 52-54°C for 1 min and extension, 72°C for 1 min) and final step at 72°C for 10 min. The 50 μl reaction mix contained 2 U of AmpliTaq Gold (Applied Biosystems), 200 μM of each dNTP and 1 μM of each primer. For details about primer pair used (position respect to BRS, sequence, amplicon length) see table below.

| **Forward** | | **Sequence (5'->3')** | **Reverse** | | **Sequence (5'->3')** | **Lenght (bp)** |
| --- | --- | --- | --- | --- | --- | --- |
| L | 103-122 | CCCTGACCCGGAGCATCTAT | H | 251-273 | GAAAAGTCTGTTAAAAGTGGTGG | 172 |
| L | 217-236 | CCCCCTTCATAAAAATTTCC | H | 400-419 | TTGGGAGACTCATCTAGGCA | 202 |
| L | 541-560 | TCAAGCACACACCCTGTAGC | H | 722-743 | TTTGGTATGGTGCTTTAACACG | 203 |
| L | 1963-1982 | TAGGCCTAAAAGCAGCCATC | H | 2162-2188 | TGGATTATTTTTATTGACTGTTAGTGG | 226 |
| L | 3249-3268 | CCGATGCAATCAAACTTTTC | H | 3442-3462 | CAGCCTGATCAGAGAATGGAG | 214 |
| L | 8124-8143 | GTGACATGCCGCAACTAGAC | H | 8309-8333 | AAAATTACAGGGGTAATAAAAGAGG | 210 |
| L | 12604-12623 | ACAGCAGCCCTACAAGCAAT | H | 12751-12771 | GGTTGCAGCTAATGCTAGTCC | 168 |
| L | 13683-13702 | GCTAGGGTATTTCCCCACAA | H | 13871-13894 | GGATTGTGATTAAGAAGGAGAGGA | 212 |

**Cloning and Sequencing**

PCR products were cloned using the TOPO TA Cloning Kit (Invitrogen) according to the manufacturer’s instructions. Screening of white recombinant colonies was accomplished by PCR, transferring the colonies into a 30 μl reaction mix (67mM Tris HCl [pH 8.8], 2 mM MgCl2 , 1μM of each primer, 0.125 mM of each dNTP, 0,75 units of Taq Polymerase) containing M13 forward and reverse universal primers. After 5 min at 92° C, 30 cycles of PCR (30 sec at 90°C, 1 min at 50°C, 1 min at 72°C) were carried out and clones with insert of the expected size were identified by agarose gel electrophoresis. After purification of these PCR products with Microcon PCR devices (Amicon), a volume of 1.5 μl was cycle-sequenced following the BigDye Terminator kit (Applied Biosystems) using the supplier’s instructions. The sequence was determined using an Applied BioSystems 3100 DNA sequencer.

**External replications**

**1.** In Trento at the TRENTO laboratory, DNA was extracted from one bone fragment with the same protocol used in Florence. Standard ancient DNA procedures for singleplex PCR amplifications, cloning and sequencing of multiple clones were followed by using the same methodology as used in Florence, with the exception that a Strata Clone TM PCR Cloning Kit was used for cloning PCRs product. Information about the six primer pairs used (position respect to BRS, sequence, amplicon length) are reported in the table below:

| **Forward** | | **Sequence (5'->3')** | **Reverse** | | **Sequence (5'->3')** | **Lenght (bp)** |
| --- | --- | --- | --- | --- | --- | --- |
| L | 541-560 | TCAAGCACACACCCTGTAGC | H | 722-743 | TTTGGTATGGTGCTTTAACACG | 203 |
| L | 2495-2515 | GGGAATGCACAAATAAGACGA | H | 2635-2654 | TCTTTAAAATCGCTCGGAGG | 160 |
| L | 12604-12623 | ACAGCAGCCCTACAAGCAAT | H | 12751-12771 | GGTTGCAGCTAATGCTAGTCC | 168 |
| L | 13150-13169 | CACAGCCTAAACGACGAACA | H | 13348-13367 | GCTGTGAAAGAGGTGGCAAT | 218 |
| L | 14093-14117 | CACAACCTCAACTTCTTTATCCTTT | H | 14269-14288 | TTTGGGGTTGATGGTCTTTT | 196 |
| L | 15310-15329 | CTCACATCAAACCCGAGTGA | H | 15501-15523 | CATGTGAGTGTCAGTAGGTCTGC | 214 |

**2.** In Uppsala, bone powder was extracted by using a modified silica-based method (Anderung et al. 2005). PCR amplifications were carried out using the same profile and condition as used in Florence with five primer pairs (see table below). PCR products were directly sequenced for both DNA strands.

| **Forward** | | **Sequence (5'->3')** | **Reverse** | | **Sequence (5'->3')** | **Lenght (bp)** |
| --- | --- | --- | --- | --- | --- | --- |
| L | 541-560 | TCAAGCACACACCCTGTAGC | H | 722-743 | TTTGGTATGGTGCTTTAACACG | 203 |
| L | 9560-9579 | TTTTGTAGCCACAGGCTTCC | H | 9744-9770 | GTTAATACTAAAAGAATAGGAGCCTCA | 211 |
| L | 12436-12459 | TCAGACCCCAATATTAACAAATTC | H | 12636-12653 | AAACCAATGTCGCCGATG | 218 |
| L | 13150-13169 | CACAGCCTAAACGACGAACA | H | 13348-13367 | GCTGTGAAAGAGGTGGCAAT | 218 |
| L | 15310-15329 | CTCACATCAAACCCGAGTGA | H | 15501-15523 | CATGTGAGTGTCAGTAGGTCTGC | 214 |

**3.** A fragment of the bone from the *Bos primigenius* specimen BVA2 was analyzed in the Australian Centre for Ancient DNA (Adelaide). Ancient DNA was extracted twice from ~500 mg powdered bone using standard proteolytic digestion and phenol/chloroform/Amicon ultra-4 purification (Shapiro et al., 2004). Two pairs of primers defining short fragments of the mitochondrial hypervariable control region and 12S-rRNA genes were used to test for the presence of amplifiable DNA using PCR (table below).

|  | **Primer** | **Primer Sequence (5’--- 3’)** | **Length^(b)^** |
| --- | --- | --- | --- |
| CR  Frag | BovCR_16738MF^(a)^ | *CACGACGTTGTAAAACGAC*ATYGTACATAGYACATTATGTCAA | ~67bp |
|  | BovCR_16810TR^(a)^ | *TACGACTCACTATAGGGCGA*GCCTAGCGGGTTGCTGGTTTCACGC |  |
| 12S  Frag | Mamm_12SE | CTATAATCGATAAACCCCGATA | ~96bp |
|  | Mamm_12SH | GCTACACCTTGACCTAAC |  |

(a): To obtain good quality sequences for short fragment from directly sequencing, M13 (CAC GAC GTT GTA AAA CGA C) and T7 (TAC GAC TCA CTA TAG GGC GA) sequence tags were added to the primers BovCR_16738F and BovCR_16810R, respectively.

(b): Length of PCR amplicon excludes primers.

(c): Primers (BovCR-16633F, BovCR-16810R, BovCR-16765F, BovCR-80R) were published in Shapiro et al., 2004.

Reactions were performed in a final volume of 25 µl containing 1 µl of aDNA extract, 1mg/ml rabbit serum albumin (RSA; Sigma, fraction V), 2 mM MgSO4, 0.6 µM of each primer, 250 µM of each dNTP, 1.25 U Platinum *Taq* Hi-Fidelity polymerase and 1 × PCR buffer (Invitrogen Ltd., UK). The thermal cycling conditions were initial denaturation at 95 °C for 2 min, followed by 50 cycles of 94 °C for 20 sec, 55 °C for 20 sec and 68 °C for 30 sec, and a final extension at 68 °C for 10 min at the end of the 50 cycles. All PCR reactions were performed on a DNA Engine Tetrad 2 Peltier Thermal Cycler (Bio-Rad). In addition, two sets of bovid-specific primers were designed to amplify an approximately 660-bp fragment of the control region in two multiplexed PCR reactions (Römpler et al., 2006). A total of 6 individual fragments were amplified in two separate reactions (A and B, Table below).

|  | **Primer** | **Primer Sequence (5' --- 3')** | **Length^(b)^** |
| --- | --- | --- | --- |
| SetA_1 | BovCR-16351F | CAACCCCCAAAGCTGAAG | ~96bp |
|  | BovCR-16457R | TGGTTRGGGTACAAAGTCTGTG |  |
| SetB_1 | BovCR-16420F | CCATAAATGCAAAGAGCCTCAYCAG | ~172bp |
|  | BovCR-16642R | TGCATGGGGCATATAATTTAATGTA |  |
| SetA_2 | BovCR-16507F | AATGCATTACCCAAACRGGG | ~184bp |
|  | BovCR-16755R | ATTAAGCTCGTGATCTARTGG |  |
| SetB_2 | BovCR-16633F^(c)^ | GCCCCATGCATATAAGCAAG | ~132bp |
|  | BovCR-16810R^(c)^ | GCCTAGCGGGTTGCTGGTTTCACGC |  |
| SetA_3 | BovCR-16765F^(c)^ | GAGCTTAAYTACCATGCCG | ~125bp |
|  | BovCR-16998R | CGAGATGTCTTATTTAAGAGGAAAGAATGG |  |
| SetB_3 | BovCR-16960F | CATCTGGTTCTTTCTTCAGGGCC | ~110bp |
|  | BovCR-80R^(c)^ | CAAGCATCCCCCAAAATAAA |  |

(a): To obtain good quality sequences for short fragment from directly sequencing, M13 (CAC GAC GTT GTA AAA CGA C) and T7 (TAC GAC TCA CTA TAG GGC GA) sequence tags were added to the primers BovCR_16738F and BovCR_16810R, respectively.

(b): Length of PCR amplicon excludes primers.

(c): Primers (BovCR-16633F, BovCR-16810R, BovCR-16765F, BovCR-80R) were published in Shapiro et al., 2004.

A multiplex PCR was performed in a final volume of 25 µl containing 2 µl of aDNA extract, 1 mg/ml rabbit serum albumin (RSA; Sigma, fraction V), 6 mM MgSO4, 0.2 µM of each primer, 500 µM of each dNTP, 2 U Platinum *Taq* Hi-Fidelity and 1 × PCR buffer (Invitrogen Ltd., UK). The multiplex PCR protocol began with an initial denaturation at 95 °C for 2 min, followed by 30 cycles of 94 °C for 15 sec, 55 °C for 20 sec and 68 °C for 30 sec, and a final extension at 68 °C for 10 min at the end of the 35 cycles. Multiplex PCR products were then diluted to 1:10 and used as a template for a second round of individual simplex PCRs, where only one primer pair was used. The simplex PCRs used Amplitaq Gold (Applied Biosystems) or Hotmaster™ *Taq* DNA polymerase (5Prime, Milton, Qld) and were performed in a final volume of 25 µl containing 1 µl of diluted multiplex PCR product, 2.5 mM MgCl2, 0.4 µM of each primer, 200 µM of each dNTP, 1 U Amplitaq Gold/ Hotmaster *Taq* polymerase and 1 × PCR buffer. The simplex PCR conditions began with an initial denaturation at 95 °C for 2 min, followed by 35 cycles of 94 °C for 20 sec, 55 °C for 15 sec and 72 °C for 30 sec, and a final extension at 72 °C for 10 min at the end of the 35 cycles. PCR products were visualised by electrophoresis and ethidium bromide staining, and PCR amplicons were purified using AMPure magnetic beads (Agencourt®, Beckman Coulter). All purified PCR products were sequenced in both directions using the ABI Prism® BigDye™ Terminator Cycle Sequencing Kit version 3.1 (Applied Biosystems). The sequencing reactions were performed in a final volume of 10 µl containing 3.2 pmol of primer, 0.25 µl Bigdye terminator premixture, and 1.875 µl of 5× sequencing buffer. The reaction conditions began with an initial denaturation at 95 °C for 2 min, 25 cycles with 95 °C for 10 sec, 55 °C for 15 sec, 60 °C for 2 min 30 sec. Sequencing products were purified using Cleanseq magnetic beads (Agencourt®, Beckman Coulter), and analyzed on an ABI 3130 DNA capillary sequencer (Applied Biosystems, Foster, CA).

**List of References**

Anderung C, Bouwman A, Persson P, Carretero JM, Ortega AI, Elburg R, Smith C, Arsuaga JL, Ellegren H, Götherström A. 2005. Prehistoric contacts over the Straits of Gibraltar indicated by genetic analysis of Iberian Bronze Age cattle. *Proc Natl Acad Sci U S A* **102**: 8431-8435.

Caramelli D, Lalueza-Fox C, Vernesi C, Lari M, Casoli A, Mallegni F, Chiarelli B, Dupanloup I, Bertranpetit J, Barbujani G. 2003. Evidence for a genetic discontinuity between Neandertals and 24,000-year-old anatomically modern Europeans. *Proc Natl Acad Sci USA* **100**: 6593–6597.

Cooper A, Poinar HN. 2000. Ancient DNA: do it right or not at all. Science **289**: 1139.

Pääbo S, Poinar H, Serre D, Jaenicke-Despres V, Hebler J, Rohland N, Kuch M, Krause J, Vigilant L, Hofreiter M. 2004. Genetic analyses from ancient DNA. *Annu Rev Genet* **38**: 645-679. Review.

Römpler H, Dear PH, Krause J, Meyer M, Rohland N, Schöneberg T, Spriggs H, Stiller M, Hofreiter M. 2006. Multiplex amplification of ancient DNA. *Nature Protocols* **1**: 720-728.

Shapiro B, Drummond AJ, Rambaut A, Wilson MC, Matheus PE, Sher AV, Pybus OG, Gilbert MT, Barnes I, Binladen J, *et al.* 2004. Rise and fall of the Beringian steppe bison. *Science* **306**: 1561-1565.

Willerslev E, Cooper A. 2005. Ancient DNA. Proc Biol Sci **272**: 3-16.
